# Subsurface chemical nanoidentification by nano-FTIR spectroscopy

**DOI:** 10.1038/s41467-020-17034-6

**Published:** 2020-07-03

**Authors:** Lars Mester, Alexander A. Govyadinov, Shu Chen, Monika Goikoetxea, Rainer Hillenbrand

**Affiliations:** 1 CIC nanoGUNE BRTA, Tolosa Hiribidea 76, 20018 Donostia-San Sebastián, Spain; 2 neaspec GmbH, Eglfinger Weg 2, 85540 Munich-Haar, Germany; 3 CIC nanoGUNE BRTA and Department of Electricity and Electronics, UPV/EHU, Tolosa Hiribidea 76, 20018 Donostia-San Sebastián, Spain; 4 IKERBASQUE, Basque Foundation for Science, 48011 Bilbao, Spain

**Keywords:** Nanoscale materials, Infrared spectroscopy, Infrared spectroscopy

## Abstract

Nano-FTIR spectroscopy based on Fourier transform infrared near-field spectroscopy allows for label-free chemical nanocharacterization of organic and inorganic composite surfaces. The potential capability for subsurface material analysis, however, is largely unexplored terrain. Here, we demonstrate nano-FTIR spectroscopy of subsurface organic layers, revealing that nano-FTIR spectra from thin surface layers differ from that of subsurface layers of the same organic material. Further, we study the correlation of various nano-FTIR peak characteristics and establish a simple and robust method for distinguishing surface from subsurface layers without the need of theoretical modeling or simulations (provided that chemically induced spectral modifications are not present). Our experimental findings are confirmed and explained by a semi-analytical model for calculating nano-FTIR spectra of multilayered organic samples. Our results are critically important for the interpretation of nano-FTIR spectra of multilayer samples, particularly to avoid that geometry-induced spectral peak shifts are explained by chemical effects.

## Abstract (web summary)

Nano-FTIR spectroscopy allows chemical characterization of composite surfaces, but its capability in subsurface analysis is not much explored. The authors show that spectra from thin surface layers differ from those of subsurface layers of the same organic material, and establish a method for distinguishing them in experiments.

## Introduction

Scattering-type scanning near-field optical microscopy (s-SNOM)^1^ is a scanning probe microscopy technique that offers nanoscale-resolved optical imaging of a wide range of samples, including polymers^2–4^, biological materials^5–8^, semiconductors^9–11^, conductors^12^ and insulators^13^. In s-SNOM, monochromatic electromagnetic radiation of the visible, infrared, or terahertz spectral range is focussed onto the tip of a standard, metallized atomic force microscope (AFM) probe. The tip—acting as an optical antenna—concentrates the radiation into highly confined and enhanced near fields at the very tip apex. The near fields interact with the sample surface, which modifies the back-scattered field in amplitude and phase, depending on the local optical sample properties. By recording the back-scattered light as a function of tip position, nanoscale-resolved images of the sample’s optical properties are obtained^1^. In order to suppress unwanted background signals, the AFM is operated in tapping mode, where the tip is oscillating normal to the sample at a frequency *Ω*. Due to the near-field interaction being strongly nonlinearly dependent on the tip-sample distance, this operation mode yields higher harmonic modulation of the tip-scattered field, but not of the background scattering. Recording the detector signal at higher harmonic frequencies *nΩ* (typically *n* > 2) thus yields the pure near-field signal^14,15^. The spatial resolution is determined by the extension of the near fields, which is in the order of the tip apex radius, which is typically around *R* = 25 nm^1,16,17^.

At infrared frequencies, s-SNOM offers the possibility for highly sensitive compositional mapping based on probing vibrational excitations such as the one of molecules or phonons, analogously to infrared microscopy^18^. Utilizing a broadband infrared source and Fourier transform spectroscopy of the light scattered by the s-SNOM tip even allows for recording nanoscale-resolved infrared spectra^19^. The technique—named nano-FTIR spectroscopy—yields near-field phase spectra that match well the absorptive properties of organic samples^19–21^, and thus allows for nanoscale chemical identification based on standard FTIR references^22^.

Despite s-SNOM and nano-FTIR being surface scanning techniques, the finite penetration depth of near fields into the sample allows for subsurface probing of nanoscale structures and defects up to a depth of 100 nm^23–27^. For s-SNOM it has been also shown that depth-resolved information—with the potential of three-dimensional sample reconstruction—can be obtained by analysis of several higher harmonic signals, each of them having a different probing depth^28–31^. However, the potential capability for chemical identification of subsurface material by nano-FTIR experiments is largely unexplored terrain.

Here we present an experimental and theoretical nano-FTIR spectroscopy study of thin subsurface organic layers. We demonstrate (1) that nano-FTIR peaks of subsurface layers are shifted to lower frequency as compared with that of bulk materials or thin surface layers, and (2) that surface and subsurface layers can be differentiated by analyzing the ratio of peak heights obtained at different demodulation orders *n*, without theoretical modeling or simulations. To that end, we have chosen to study, exemplarily, the well-defined C=O vibrational mode of a thin polymethyl-methacrylate (PMMA) layer on silicon covered by a polystyrene (PS) layer of varying thickness, which we compare with differently thick uncovered PMMA layers on silicon. We further elucidate how a semi-analytical model can be used to understand and predict nano-FTIR spectra of multilayered samples. We finally demonstrate validity and applicability of our findings for a large variety of materials, by summarizing and discussing the results of an extended theoretical and experimental study of the nano-FTIR peak characteristics of differently thick subsurface layers exhibiting various molecular vibrational modes.

## Results

### Experimental nano-FTIR study of subsurface organic material

**Fig. 1:**
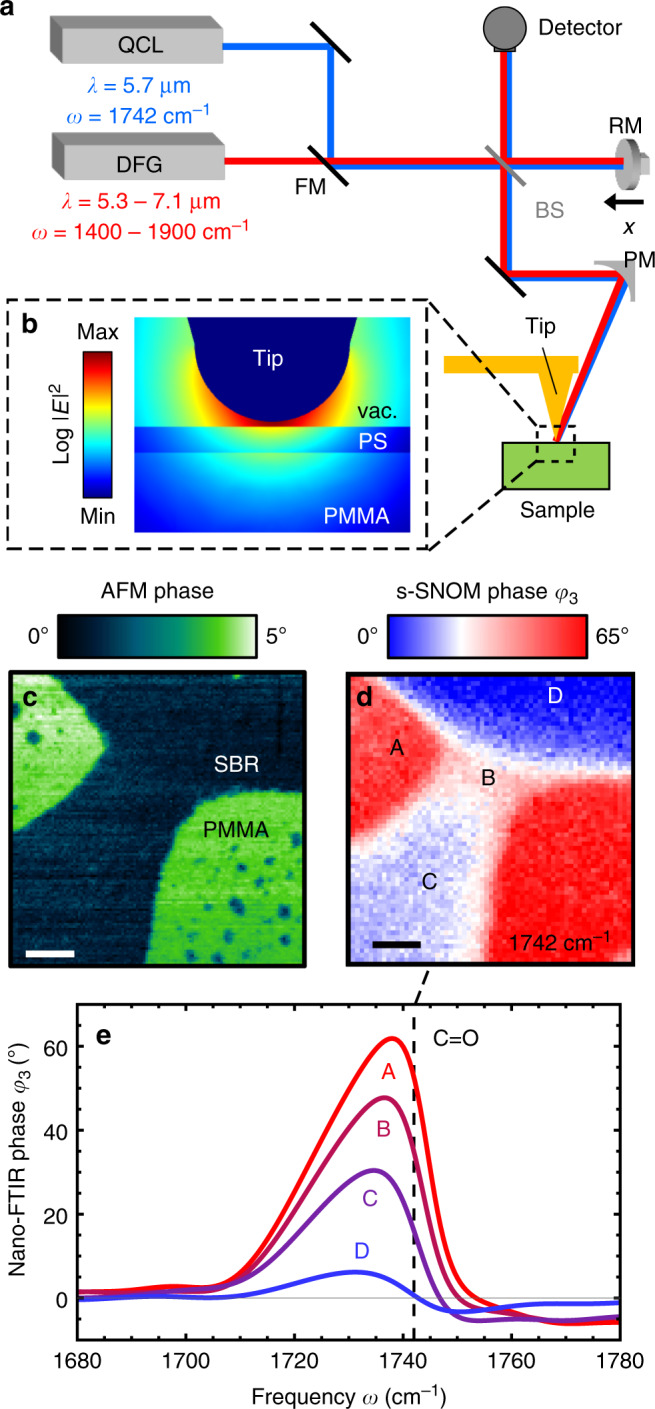
Near-field imaging and spectroscopy of organic nanocomposites. **a** Illustration of the s-SNOM and nano-FTIR setup. A quantum cascade laser (QCL) is used for s-SNOM imaging. An infrared laser continuum based on difference frequency generation (DFG) is used for nano-FTIR spectroscopy. The light source is selected with a flip mirror (FM). A parabolic mirror (PM) is used for focussing the laser radiation onto the tip apex. After collection of the tip-scattered light with the PM, a Michelson interferometer comprising a beam splitter (BS) and moveable reference mirror (RM) is used for detection. **b** Simulated near-field distribution around a tip apex (30 nm radius) above a 10 nm-thick PS layer on PMMA. For simulation details see “Methods” section. **c** AFM mechanical phase image of a two-component rubber blend (SBR/PMMA) and **d** corresponding s-SNOM phase *φ*_3_ image recorded at 1742 cm^−1^, which maps the absorption of the C=O vibrational mode of PMMA. Scale bar: 200 nm. **e** Nano-FTIR phase *φ*_3_ spectra of selected positions A–D. Vertical dashed line marks the imaging frequency.

For subsurface infrared near-field spectroscopy we use a setup based on a commerical s-SNOM (neaSNOM from neaspec GmbH), which employs a tuneable quantum cascade laser (Daylight Solutions) for s-SNOM imaging and a broadband infrared laser continuum (generated by difference frequency generation) for nano-FTIR spectroscopy (Fig. 1a). The laser beams are focussed onto a standard platinum-coated AFM tip (NCPt arrow tip, Nanoworld), which is in close proximity to the sample. The AFM tip acts as an optical antenna and creates strongly enhanced near fields around the tip apex, yielding a spatial resolution in the order of the tip radius, here *R* = 25 nm. Importantly, the near fields penetrate into the sample (Fig. 1b), thus allowing for probing of subsurface material. For background suppression, the AFM is operated in tapping mode. We utilize a tip oscillation amplitude *A* = 30 nm, frequency *Ω* = 230 kHz and demodulation orders *n* = 3 and *n* = 4. Detection of the tip-scattered light in an asymmetric Fourier transform spectrometer yields the complex-valued spectral scattering coefficient $\sigma _n = s_n{\mathrm{e}}^{{\mathrm{i}}\varphi _n}$, which can be separated into near-field (nano-FTIR) amplitude *s*_*n*_ and phase *φ*_*n*_ spectra. For a quantitative analysis, all spectra are normalized to the nano-FTIR spectrum of a clean silicon substrate via $\sigma _n^{{\mathrm{norm}}} = \sigma _n/\sigma _n^{{\mathrm{Si}}}$ . We focus our analysis on organic sample systems and thus evaluate normalized nano-FTIR phase spectra $\varphi _n^{{\mathrm{norm}}}(\omega ) = {\mathrm{Arg}}[\sigma _n/\sigma _n^{{\mathrm{Si}}}]$, which qualitatively relate to the absorptive properties of molecular samples^2,5,32,33^. The superscript ^norm^ is omitted in the following for simplicity.

We motivate our systematic nano-FTIR spectroscopy study of well-defined multilayer samples with s-SNOM images and nano-FTIR spectra of a styrene-butadiene rubber (SBR)/PMMA composite sample of unknown vertical structure. Figure 1c shows the AFM mechanical phase image, revealing two homogeneous areas that indicate a separation of PMMA and SBR with sharp boundaries at the sample surface. To map the absorption of the C=O vibrational mode of PMMA, we recorded an s-SNOM phase image of the same area at *ω* = 1742 cm^−1^ (Fig. 1d). We find two strongly absorbing areas (red, marked A), corresponding to the green areas in the mechanical phase image (Fig. 1c) that subsequently can be identified as PMMA (note that SBR has no absorption in the spectral region of the C=O bond^34^). Interestingly, we find significant s-SNOM phase signals within the SBR area, indicating the presence of PMMA. Considering the sharp material boundaries observed in the mechanical phase images, we assume that PMMA is located below the SBR. The presence of PMMA is confirmed by nano-FTIR spectra recorded at positions B–D, which clearly reveal the same spectral peak as observed at position A (Fig. 1e). However, we observe a significant shift of the peak to lower frequencies when the peak maximum decreases. As we can exclude peak shifts due to chemical interaction^35,36^, we speculate that the peak shifts are due to the subsurface location of PMMA. To corroborate subsurface nano-FTIR spectroscopy of organic materials and to confirm that it comes along with significant peak shifts, we performed a fundamental comparative study of multilayer organic samples with well-defined composition and geometry, as described in the following.

**Fig. 2:**
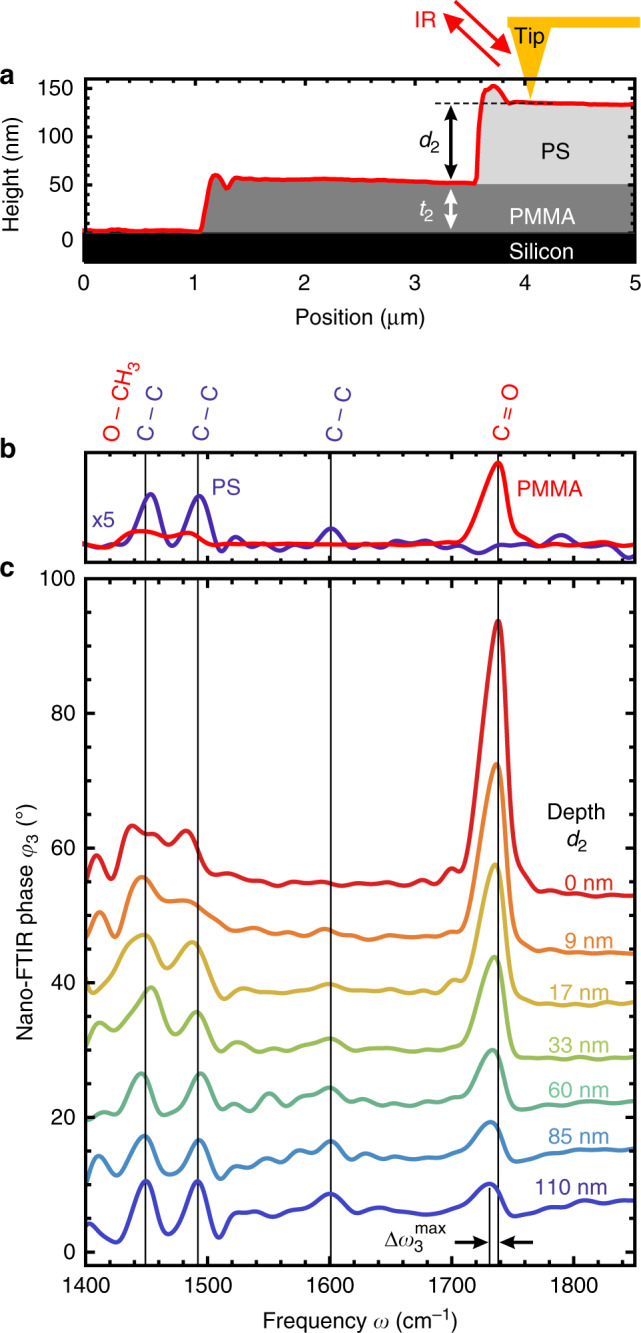
Subsurface nano-FTIR spectroscopy experiments on well-defined multilayer samples. **a** Schematics of the experiment and PMMA/PS test sample, including the topography line profile of a *d*_2_ = 85 nm-thick PS layer covering the *t*_2_ = 59.4 nm-thick PMMA layer on Si. **b** Reference nano-FTIR phase spectra recorded on thick PMMA and PS layers. **c** Subsurface nano-FTIR phase spectra of PMMA at different depths *d*_2_ below PS (average of 50 spectra (80 spectra for *d*_2_ ≥ 85 nm), 30 s acquisition time per interferogram, ×128 zero filling, 17 cm^−1^ spectral resolution). Black arrows in **c** indicate the spectral peak shift ${\mathrm{\Delta }}\omega _3^{{\mathrm{max}}}$.

As model sample for subsurface nano-FTIR spectroscopy we have chosen a PMMA layer of thickness *t*_2_ = 59.4 ± 4.7 nm on a silicon substrate that is covered by a PS layer of varying thickness *d*_2_ = 0–110 nm (see schematics and AFM line profile in Fig. 2a, for fabrication details see “Methods”). Reference nano-FTIR phase spectra of PMMA and PS are shown in Fig. 2b. We identify PMMA via the C=O vibrational stretch mode around $1738\, {\mathrm{cm}}^{ - 1}$^37^. The smaller plateau-like feature around 1440–1500 cm^−1^ corresponds to vibrations in the O−CH_3_ group. Characteristic for PS are the two distinct absorption lines at 1452 cm^−1^ and 1493 cm^−1^ (and a weaker mode at 1601 cm^−1^), which arise from C−C stretching vibrations in the aromatic ring^38^. The reference spectrum of PS does not exhibit any phase contrast around 1700–1800 cm^−1^, i.e., it is spectrally flat, which simplifies the following discussion.

Figure 2c shows subsurface nano-FTIR spectra of PMMA at different depths *d*_2_ below PS. Without a capping layer (red, *d*_2_ = 0), we clearly observe the characteristic C=O and O−CH_3_ vibrational modes of PMMA. As the depth *d*_2_ of the PMMA layer (i.e., thickness *t*_1_ of the PS capping layer) increases, the spectral feature from 1440–1500 cm^−1^ gradually changes from plateau-like (which is characteristic for PMMA) toward two distinct peaks at 1452 cm^−1^ and 1493 cm^−1^ (which are characteristic for PS). Simultaneously, the C=O peak height (PMMA) rapidly decreases with depth *d*_2_, but still allows for chemical identification of PMMA at a depth of *d*_2_ = 110 nm. We note that the C=O peak shifts to lower frequencies (red shifts) with increasing depth (indicated in Fig. 2c by ${\mathrm{\Delta }}\omega _3^{{\mathrm{max}}}$), which reminds us of peak shifts previously reported in experimental nano-FTIR studies of surface PMMA layers^22^ and surface silicon dioxide layers^39^ of varying thicknesses.

**Fig. 3:**
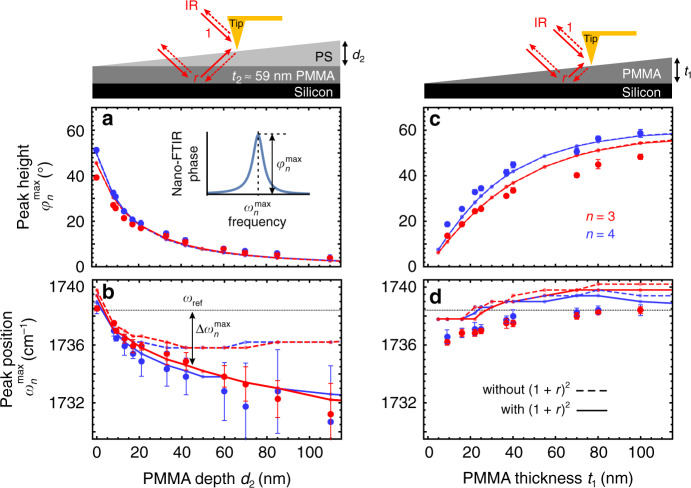
Comparison of PMMA subsurface layers and thin PMMA surface layers. **a**, **c** Peak heights $\varphi _n^{{\mathrm{max}}}$ and **b**, **d** spectral peak positions $\omega _n^{{\mathrm{max}}}$ extracted from experimental (large dots) and calculated (lines) nano-FTIR phase spectra, as a function of PMMA depth *d*_2_ (left column) and PMMA thickness *t*_1_ (right column), for different demodulation orders *n* = 3 (red) and *n* = 4 (blue). The solid and dashed lines are calculated with and without the far-field factor (1 + *r*)² in Eq. (1), respectively. The gray horizontal dotted line indicates the peak position *ω*_ref_ experimentally obtained on thick (bulk) PMMA samples. Inset: definition of $\varphi _n^{{\mathrm{max}}}$ and $\omega _n^{{\mathrm{max}}}$. Each experimental data point shows mean values and standard deviation (error bars) obtained from ten averaged (5×) spectra (16 averaged spectra for *d*_2_ ≥ 85 nm).

In order to better understand the nano-FTIR phase spectra, we focus our analysis on the C=O peak of PMMA around 1738 cm^−1^. Specifically, we investigate the depth dependence of the spectral peak position $\omega _n^{{\mathrm{max}}}$ and peak height $\varphi _n^{{\mathrm{max}}}$ (defined in the inset of Fig. 3) for the two different higher harmonic demodulation orders *n* *=* 3 and *n* = 4. For comparison, we performed a similar study on thin PMMA layers of varying thickness *t*_1_ at the surface. The results are shown in Fig. 3 (large symbols).

Figure 3a, b shows the results for the subsurface PMMA layer. We observe that the peak height (Fig. 3a) for both demodulation orders rapidly decreases when the depth *d*_2_ of the PMMA layer (i.e., thickness of the PS capping layer) increases. This decay can be explained by the exponential decay of the near fields from the tip apex into the sample (as seen in Fig. 1b). The deeper the PMMA layer, the less near fields it is absorbing, i.e., the less material is probed. The C=O peak can still be identified at a depth of *d*_2_ = 110 nm. Further, we observe a red shift of the peak position with respect to *ω*_ref_ (horizontal line in Fig. 3b) with increasing *d*_2_ (of up to $\Delta \omega _3^{{\mathrm{max}}} = \omega _{{\mathrm{ref}}} - \omega _3^{{\mathrm{max}}} = 7\, {\mathrm{cm}}^{ - 1}$ at *d*_2_ = 110 nm). Interestingly, we find that signal demodulation at higher harmonics yields stronger red shifts and a faster decay of the peak height with increasing depth, that is, the relative contrast $C = \varphi _4^{{\mathrm{max}}}/\varphi _3^{{\mathrm{max}}}$ decreases with increasing depth. We attribute this behavior to the stronger confinement of near fields to the tip apex at higher demodulation orders^23,30,40^. We further analyze *C* below to distinguish nano-FTIR spectra of subsurface layers from thin layers at the surface.

For comparison, Fig. 3c, d shows the results for the surface PMMA layers of varying thickness *t*_1_. We observe that the peak height $\varphi _n^{{\mathrm{max}}}$ for both demodulation orders slowly decreases when the thickness *t*_1_ of the PMMA layer decreases. This decay can be explained by the decreased amount of absorbing material within the nano-FTIR probing volume. The thinner the PMMA layer, the less near fields it is absorbing, i.e., more silicon is probed, which is non-absorbing in this spectral range. Further, we see a red shift of the peak position with decreasing *t*_1_ (of up to $\Delta \omega _3^{{\mathrm{max}}} = 2\ {\mathrm{cm}}^{ - 1}$), as previously reported and explained by Mastel et al.^22^. A thin organic layer on a highly reflective substrate promotes reflections of near fields between the tip and sample, which changes the probing mechanism from absorption-like to an absorption-reflection mechanism, thus causing a shift of the peak position^22^. Interestingly, Fig. 3 also shows that the spectral peak shift in nano-FTIR phase spectra is much larger for subsurface layers when compared with surface layers. Notably, and in contrast to the subsurface PMMA layers, we find that the relative contrast $C = \varphi _4^{{\mathrm{max}}}/\varphi _3^{{\mathrm{max}}}$ and the spectral peak position for PMMA layers at the sample surface vary only slightly with the demodulation order *n*, as we further analyze and exploit below.

### Interpretation of nano-FTIR spectra of multilayered samples

**Fig. 4:**
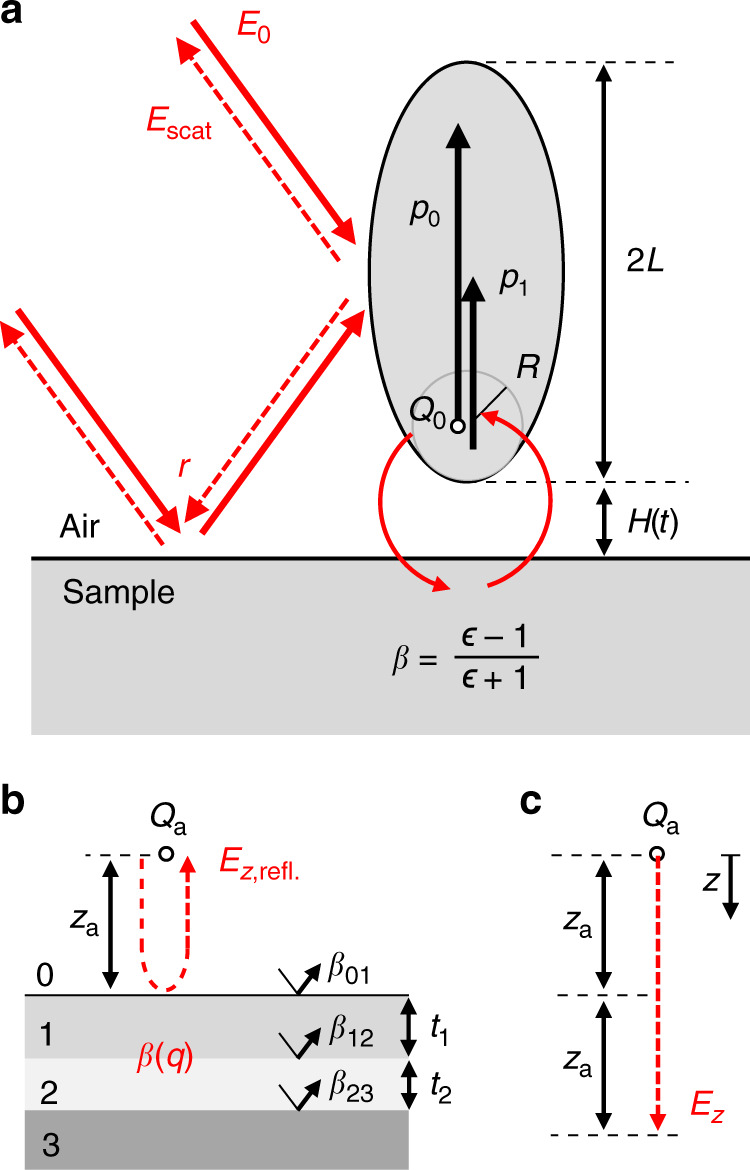
Illustration of the finite dipole model for bulk and multilayered samples. **a** The nano-FTIR tip is modeled as a prolate spheroid of length 2*L* and apex radius *R*, which is located at height *H*(*t*) above a bulk sample with permittivity *ϵ* and electrostatic reflection coefficient *β* = (*ϵ* *−* 1)/(*ϵ* *−* 1). The incident electric field *E*_0_ induces the primary electric dipole *p*_0_, which interacts with the sample via the point charge *Q*_0_, yielding the near-field induced dipole *p*_1_ (indicated by red curved arrows). The model accounts for far-field illumination and detection of the tip-scattered field *E*_scat_ via reflection at the sample surface, described by the Fresnel reflection coefficient *r* (indicated by red straight arrows). Illustration of (**b**) monopole field reflected at a multilayer sample, *E*_*z*,refl_, and (**c**) monopole field *E*_*z*_ without sample. Both red dashed arrows have a length of 2*z*_a_. The multilayer sample in **b** is characterized by the quasi-electrostatic reflection coefficient *β*(*q*), which is obtained from the single-interface electrostatic reflection coefficients *β*_*ij*_ and layer thicknesses *t*_*j*_.

To better understand nano-FTIR spectra of vertically inhomogeneous samples, we performed model calculations based on the finite dipole model (FDM), which is illustrated in Fig. 4a and explained in more detail in the Supplementary methods. In short, s-SNOM signals are described by calculating the scattering coefficient *σ* = *E*_scat_/*E*_0_, where *E*_0_ is the incident field and *E*_scat_ is the tip-scattered field. The tip is modeled as elongated spheroid with major half-axis length *L* and tip apex radius *R*. It is illuminated directly and indirectly via reflection at the sample surface with the far-field reflection coefficient *r*, yielding a local electric field at the tip, $E_{{\mathrm{loc}}} \propto \left( {1 + r} \right)E_0$, which induces an electric dipole *p*_0_ in the tip. The near-field interaction of *p*_0_ with the sample is mediated predominantly via one of the charges associated with this dipole, *Q*_0_, located close to the tip apex^15,41^, which induces an additional dipole *p*_1_ in the tip. The total induced dipole moment is *p* = *p*_0_ + *p*_1_ = *α*_eff_*E*_loc_, where *α*_eff_ is the effective polarizability of the coupled tip-sample system. The scattered (far) field of this dipole is measured directly and via reflection from the sample, *E*_scat_ ∝ (1 + *r*)*p*. The scattering coefficient can thus be described by:

1 $$\sigma = \left( {1 + r} \right)^2\alpha _{{\mathrm{eff}}} \cdot$$

In the FDM, the effective polarizability is given by^15,41^:

2 $$\alpha _{{\mathrm{eff}}} = 1 + \frac{1}{2}\frac{{f_0(H)\beta ({\it{\epsilon }})}}{{1 - f_1(H)\beta ({\it{\epsilon }})}},$$

where *β* = (*ϵ* *−* 1)/(*ϵ* + 1) is the quasi-electrostatic reflection coefficient of a semi-infinite (bulk) sample with permittivity *ϵ*, and *f*_*i*_(*H*) (given in Supplementary methods) describe the tip geometry and tip-sample distance *H*(*t*) = *A*(1 + cos *Ωt*). The higher harmonic signal demodulation (used for background suppression) is included in the model by taking the *n*th Fourier coefficient $\hat F_n$ with respect to time, yielding the *n*th-order demodulated scattering coefficient^15,41^:

3 $$\sigma _n = \left( {1 + r} \right)^2\hat F_n\left[ {1 + \frac{1}{2}\frac{{f_0\left( {H\left( t \right)} \right)\beta }}{{1 - f_1\left( {H\left( t \right)} \right)\beta }}} \right].$$

To facilitate the interpretation of Eq. (3) in the following, we express the scattering coefficient as Taylor series in *β* of order *N*^42^, such that nano-FTIR signals are simply proportional to powers *j* of *β*:

4 $$\sigma _n = \left( {1 + r} \right)^2 \cdot \frac{1}{2}\sum_{j = 1}^N {\hat F_n\left[ {f_0f_1^{j - 1} \cdot \beta ^j} \right]} ,$$

which is valid for weak molecular oscillators with |*β*| < 1 (which includes for example polymers and biological matter).

Equations (3) and (4) can be used to calculate relative material contrasts in nano-FTIR spectroscopy, however, they are valid only for semi-infinite samples. To extend the model to multilayered samples, we use the multilayer reflection coefficient *β*(*q*) which depends on the momentum *q* and for one layer on a substrate it is given by^22,43^:

5 $$\beta \left( q \right) = \frac{{\beta _{01} + \beta _{12}{\mathrm{e}}^{ - 2qt_1}}}{{1 + \beta _{01}\beta _{12}{\mathrm{e}}^{ - 2qt_1}}},$$

where *β*_*ij*_ = (*ϵ*_*j*_ − *ϵ*_*i*_)/(*ϵ*_*j*_ + *ϵ*_*i*_) are the single-interface reflection coefficients and *t*_1_ is the layer thickness, as indicated in Fig. 4b. Multiple layers on a substrate can be described by recursively using Eq. (5) as expression for *β*_12_^44^. The FDM (Eqs. (3) and (4)), however, does not explicitly support momentum-dependent reflections (because the quasi-static approximation *q* → ∞ is made). For that reason, we substitute *β* in Eqs. (3) and (4) by the effective near-field reflection coefficient $\bar \beta = E_{{\it{z}},{\mathrm{refl}}}/E_{{\it{z}}}$, where *E*_*z*,refl_ is the electric field produced by an effective point charge *Q*_a_ (located at a distance *z*_a_ from the sample surface, Fig. 4b), reflected at the multilayered sample surface and evaluated at the position of *Q*_a_ itself (indicated by red dashed arrow in Fig. 4b). *E*_*z*_ is the electric field of *Q*_a_ at a distance of 2*z*_a_ (Fig. 4c). It can be shown (see Supplementary methods) that:

6 $$\bar \beta = \frac{{E_{{z},{\mathrm{refl}}}}}{{E_z}} = \frac{{\mathop {\smallint }\nolimits_0^\infty \beta \left( q \right)q\rm{e}^{ - 2{\it{q}{z}}_{a}}\it{dq}}}{{\mathop {\smallint }\nolimits_0^\infty q\rm{e}^{ - 2{\it{q}}{\it{z}}_a}\it{dq}}},$$

where *z*_a_ = *H*(*t*) + *a*. Here *a* describes the height of the effective charge *Q*_a_ above the tip apex. The value *a* will be found such that good agreement between calculation and experiment is achieved.

We use Eqs. (4) and (6) to calculate nano-FTIR phase spectra *φ*_*n*_(*ω*) of surface and subsurface PMMA layers corresponding to the sample geometries of Fig. 3 and extract the spectral C=O peak positions $\omega _n^{{\mathrm{max}}}$ and peak heights $\varphi _n^{{\mathrm{max}}}$ for various layer thicknesses and depths (Supplementary Fig. 2). PMMA is described by dielectric permittivity data obtained by infrared ellipsometry^45^ and we assume PS and Silicon to be non-absorbing in the considered spectral range, with *ϵ*_PS_ = 2.5 and *ϵ*_Si_ = 11.7^46,47^. For the tapping amplitude *A* and tip radius *R* we use the experimental values *A* = 30 nm and *R* = 30 nm. Convergence of the calculated $\omega _n^{{\mathrm{max}}}$ and $\varphi _n^{{\mathrm{max}}}$ (red and blue lines for demodulation orders *n* = 3 and *n* = 4, respectively) is achieved for Taylor expansion orders *N* ≥ 5, and we find good agreement with the experimental data for *a* = 1.4*R*. The effective charge *Q*_a_ is thus located slightly higher than the charge *Q*_0_ (located at distance *R* from the tip apex), indicating that the near-field interaction takes place also via the apex-near part of the tip shaft, which is located further away than the tip apex. Indeed, probing of subsurface layers is frequently attributed to the elongated tip shape, which provides longer-reaching evanescent waves (with lower momenta *q*) that are not captured well by the unmodified FDM for bulk samples^25,39,43^.

Note that for large subsurface layer depths *d*_2_ > 30 nm, we observe a dispersive line shape of the nano-FTIR peaks (Fig. 2c). We attribute this finding to the far-field reflection of both the illumination and tip-scattered field at the sample surface (considered in Eq. (1) by the far-field refection coefficient *r*), which carries the far-field absorption characteristics of the subsurface PMMA layer. This PMMA far-field contribution to the tip-scattered field becomes notable when the near-field contribution vanishes at larger probing depths. We support our explanation with Supplementary Fig. 3, where we compare calculated nano-FTIR peak shapes, revealing that the far-field factor (1 + *r*)² in Eq. (1) indeed yields a dispersive line shape for large *d*_2_. For a more quantitative comparison, we show in Fig. 3 the calculated peak positions and peak heights obtained without the far-field reflection coefficient (dashed lines). We find that for *d*_2_ < 20 nm the peak positions are nearly the same as for the calculation including the factor (1 + *r*)² (solid lines), revealing that the peak shift is essentially a near-field effect. For *d*_2_ > 20 nm, the peak shift stays rather constant and clearly differs from the calculation including the factor (1 + *r*)². The continuous red shift for *d*_2_ > 20 nm observed in the experiment can be thus attributed to the far-field contribution. We note that the constant peak position for *d*_2_ > 20 nm does not imply the absence of near-field probing. The peak height still decreases until *d*_2_ > 100 nm, which is a clear near-field signature. We finally note that for layers of lateral extensions smaller than the illumination wavelength we expect the contribution from the far-field reflection becoming less pronounced, yielding peak shifts located between the dashed and solid curves in Fig. 3.

The analytical nature of our model lets us elucidate the physical cause of the spectral peak shifts observed in nano-FTIR phase spectra of multilayered samples. Our model (Eqs. (4) and (6)) shows that the spectral behavior of nano-FTIR signals essentially follows the spectral behavior of $\bar \beta$, as neither the exponent *j* nor the geometry factors *f*_0_ and *f*_1_ in Eq. (4) lead to spectral peak shifts. Furthermore, $\bar \beta$ can be interpreted as weighted average of *β*(*q*), with the weights being determined by the (spectrally independent) coupling weight function (CWF):

7 $$w\left( {q,H} \right) = q\rm{e}^{ - 2{\it{q}}{\it{z}}_a(\it{H})}.$$

**Fig. 5:**
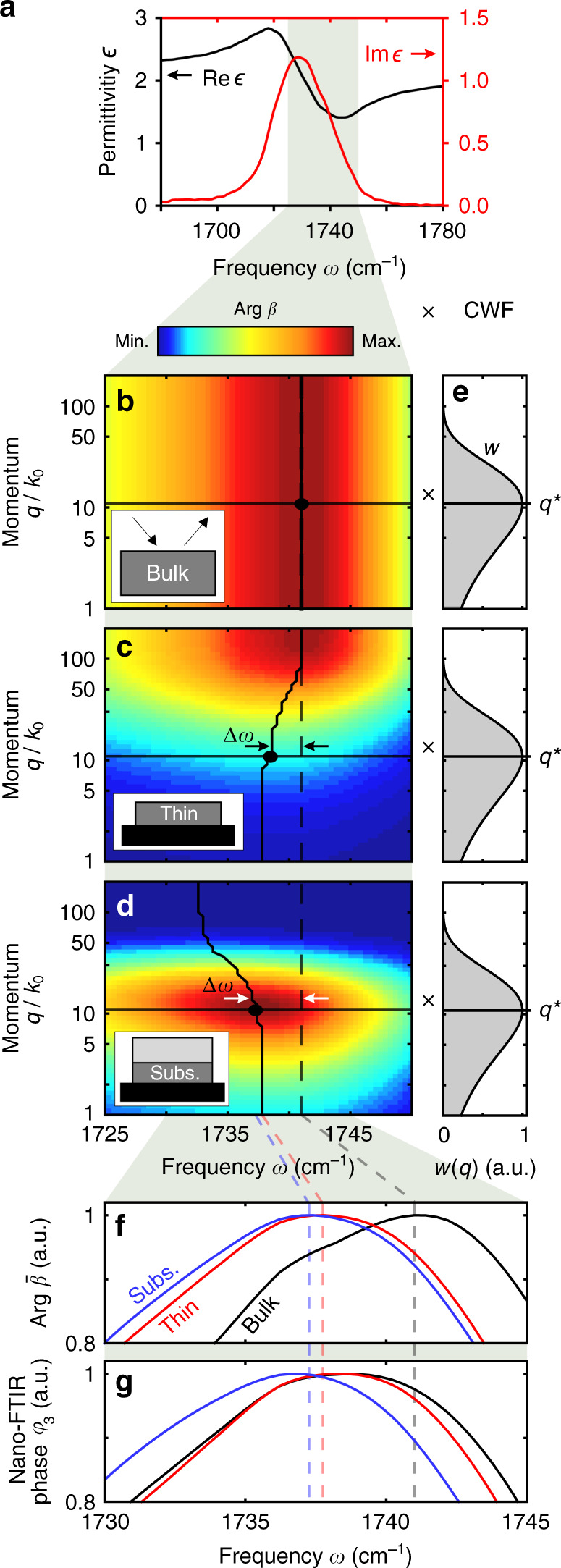
Geometry- and momentum-dependent spectral shifts in nano-FTIR phase spectra. **a** Real (black) and imaginary (red) parts of the dielectric function of PMMA, showing a typical Lorentz oscillator shape. Phase of the Fresnel reflection coefficient, Arg *β*, for different PMMA samples: **b** bulk PMMA, **c** *t*_1_ = 20 nm-thick PMMA layer at the surface of a silicon substrate, **d** *t*_2_ = 20 nm-thick subsurface PMMA layer at the surface of a silicon substrate and at depth *d*_2_ = 40 nm below PS. The vertical/curved solid lines indicate the frequency where Arg *β* has its maximum. **e** Coupling weight function *w*(*q*) calculated with the tip in contact with sample (*H* = 0). Horizontal lines indicate the momentum q* of highest tip-sample coupling. **f** Phase of near-field reflection coefficient, Arg $\bar \beta$, obtained by multiplying the Fresnel reflection coefficient (panels **b**–**d**) with the CWF (panel **e**), and integration over all momenta *q*. **g** Calculated nano-FTIR phase spectra *φ*_3_.

To illustrate the spectral shifts, we compare in Fig. 5 the phase of the Fresnel reflection coefficient, Arg *β*(*ω*,*q*), for three PMMA samples with different geometries. The C=O vibrational mode of PMMA is described by a dielectric function (plotted in Fig. 5a), which can be well described by a Lorentz oscillator. It can be thus considered a highly representative example of a typical molecular vibration. Figure 5b shows Arg *β*(*ω*,*q*) of bulk PMMA. We find a spectral maximum (traced by the vertical black line) at 1741 cm^−1^, which is independent of *q*. The situation changes for a thin PMMA layer (thickness *t*_1_ = 20 nm) on a silicon substrate (Fig. 5c). For decreasing *q*, we observe that the spectral maximum shifts to lower frequencies when compared with bulk PMMA (as indicated by Δ*ω* in Fig. 5c). Analysing finally Arg *β*(*ω*,*q*) for a subsurface PMMA layer (thickness *t*_2_ = 20 nm, on top of Si substrate and covered by a *d*_2_ = 40 nm-thick PS layer), we find that for all momenta *q* the spectral maximum is shifted to lower frequencies compared with bulk PMMA (Fig. 5d). This shift Δ*ω* is larger than that of thin PMMA surface layers (Fig. 5c) and increases with increasing *q* (contrary to the thin surface layer). For a comparison with nano-FTIR phase spectra *φ*_*n*_(*ω*) we have to consider that the nano-FTIR tip provides and probes a broad distribution of momenta, which is described by the CWF, i.e., for a tip-sample distance *H* = 0 (Fig. 5e). Note that similar expressions for a CWF are also found in mathematically rigorous s-SNOM models^43,48,49^, however a direct correlation with nano-FTIR spectra is less apparent due to the complexity of such models. Here, we multiply the CWF (Fig. 5e) with the Fresnel reflection coefficient (Fig. 5b–d) and subsequently integrate over all momenta *q*, and we obtain Arg $\bar \beta (\omega )$ (Fig. 5f), which we directly compare with calculated nano-FTIR phase spectra *φ*_3_(*ω*) (Fig. 5g). We find qualitative agreement between the spectral shifts in Arg $\bar \beta (\omega )$ and *φ*_3_(*ω*), indicating that the root cause of spectral nano-FTIR shifts in layered samples are spectral shifts of the multilayer Fresnel reflection coefficient and thus a true spectroscopic feature of the sample.

The quantitative differences between Arg $\bar \beta (\omega )$ and *φ*_3_(*ω*) can be explained by signal demodulation in nano-FTIR, which has not been considered so far in our analysis of $\bar \beta$. Eqs. (4) and (6) imply that signal demodulation (described by the Fourier components $\hat F_n$) acts on the CWF, *w*(*q, H*), which depends on the modulated tip-sample distance *H*. It is well known^23,29,30^ that s-SNOM imaging at higher demodulation orders improves the lateral spatial resolution and reduces the probing depths, indicating that near fields of larger momenta are probed. This corresponds to near-field probing at higher momenta *q* for increasing *n*, and explains the reduction of the nano-FTIR spectral peak shifts for surface layer and an increase for subsurface layers (compared with bulk) observed in Fig. 5g. As probing with higher momenta implies better spatial resolution, we expect that inhomogeneities in surface layers can be better resolved than in subsurface layers.

We point out that the observed nano-FTIR peak shifts are not specific to near-field spectroscopy. They also occur in far-field infrared spectroscopy, for example, when the reflected power $R = \left| {r\left( {\omega ,q} \right)} \right|^2$ is measured^50^. This is because the far-field reflection coefficient *r*(*ω,q*) depends—as well as the quasi-electrostatic near-field reflection coefficient—on the momentum *q* = *k*_0_sin(*Θ*), which in far-field spectroscopy is determined by angle of incidence, *Θ*. We illustrate this dependence and the resulting peak shifts by comparing normal- and grazing-incidence reflection spectra (*Θ* = 0° and 80°, respectively) of a thin PMMA layer in Supplementary Fig. 4, which are shifted by 10 cm^−1^.

### Model-free differentiation of subsurface and surface layers

**Fig. 6:**
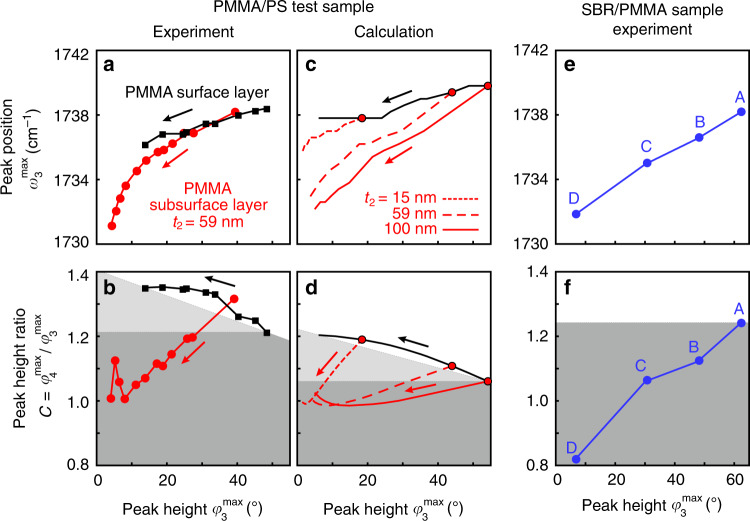
Correlation of nano-FTIR peak characteristics. **a** Spectral peak positions $\omega _3^{{\mathrm{max}}}$ and **b** peak height ratios *C* of PMMA surface (black symbols) and PMMA subsurface (red symbols) layers are plotted versus the corresponding peak heights $\varphi _3^{{\mathrm{max}}}$ (experimental data taken from Fig. 3). Arrows indicate decreasing PMMA surface layer thickness *t*_1_ (black) and increasing PMMA subsurface layer depth *d*_2_ (red). Subsurface PMMA layer thickness is *t*_2_ = 59 nm. **c**, **d** Calculation results analogous to Fig. 6a, b. In addition, results for PMMA subsurface layers of thicknesses *t*_2_ = 15 nm (dotted red line) and *t*_2_ = 100 nm (solid red line) are shown. **e** PMMA nano-FTIR peak positions $\omega _3^{{\mathrm{max}}}$ and **f** peak height ratios *C* are plotted versus the corresponding peak heights $\varphi _3^{{\mathrm{max}}}$ (data measured at positions A–D in Fig. 1d). **b**, **d**, **f** Gray areas indicate the data spaces that correspond to subsurface material.

**Fig. 7:**
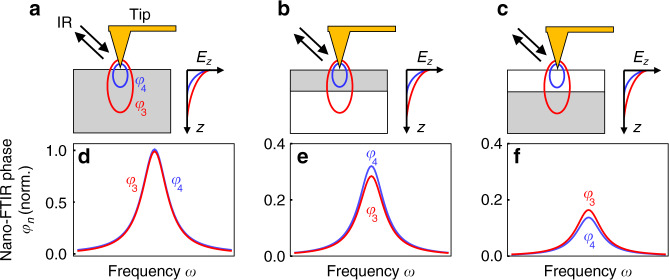
Illustration of the peak height ratio criterium. **a**–**c** Schematic of the nano-FTIR experiment employing different higher harmonic demodulation orders *n* = 3 (red) and *n* = 4 (blue) for three samples with different geometries: **a** bulk material, **b** thin surface layers, and **c** subsurface layers. Gray areas mark the absorbing material. Ellipses below the tip illustrate the probing volumes. Graphs illustrate the exponential decay of near fields *E*_*z*_ for different demodulation orders. **d**–**f** Sketch of qualitative nano-FTIR phase spectra, corresponding to panels **a**–**c**. For a given *n*, all *φ*_*n*_ spectra are normalized to $\varphi _n^{{\mathrm{max}}}$ of bulk.

Finally, we elucidate how the observed spectral differences between nano-FTIR spectra of thin organic layers at the surface and subsurface organic layers could be exploited for distinguishing the two cases without the need of theoretical modeling, which is to date still a challenging and time-consuming task. To this end, we analyze the correlation between peak height $\varphi _3^{{\mathrm{max}}}$ and spectral peak position $\omega _3^{{\mathrm{max}}}$, as well as the correlation between peak height $\varphi _3^{{\mathrm{max}}}$ and peak height ratio $C = \varphi _4^{{\mathrm{max}}}/\varphi _3^{{\mathrm{max}}}$. In Fig. 6a, c (experimental and calculated data, respectively), we observe that the peak position in nano-FTIR phase spectra of both thin PMMA surface layers (black symbols) and PMMA subsurface layers (red symbols) show similar trends, i.e., that a decrease in peak height comes along with a spectral red shift. However, for subsurface layers, the red shift is much stronger. The peak height ratio $C = \varphi _4^{{\mathrm{max}}}/\varphi _3^{{\mathrm{max}}}$ shows opposing trends for thin PMMA surface and subsurface layers (experimental and calculated data in Fig. 6b, d, respectively). With decreasing peak height, we find that *C* increases for surface layers and decreases for subsurface layers. We explain this correlation of *C* and peak height by the reduced probing depth (and increased surface sensitivity) at higher harmonic demodulation orders^23,30,40^. The reduced probing depth at higher harmonic demodulation orders (illustrated in Fig. 7) causes $\varphi _4^{{\mathrm{max}}}$ to decrease faster than $\varphi _3^{{\mathrm{max}}}$, as the depth *d*_2_ of a subsurface layer increases—thus the ratio $\varphi _4^{{\mathrm{max}}}/\varphi _3^{{\mathrm{max}}}$ decreases for subsurface layers (Fig. 7c, f). On the other hand, as the thickness *t*_1_ of a surface layer decreases, the increased surface sensitivity at higher demodulation orders causes $\varphi _4^{{\mathrm{max}}}$ to reduce slower than $\varphi _3^{{\mathrm{max}}}$—thus the ratio $\varphi _4^{{\mathrm{max}}}/\varphi _3^{{\mathrm{max}}}$ increases for surface layers (Fig. 7b, e).

In the calculations we also considered subsurface layers of different thicknesses *t*_2_. We find that both the peak shifts and peak height ratios (red curves in Fig. 6c and d, respectively) are always located below the curves obtained for the surface layers (black lines). Our observations thus offer a rather easy procedure for distinguishing surface and subsurface layers. We assume that the correlations for PMMA surface layers (black curves) are known from reference measurements or can be approximated by a straight line connecting reference measurements obtained for a very thin surface layer (leftmost black data points in Fig. 6b, d) and a bulk surface (rightmost black data points in Fig. 6b, d). Sample measurements yielding data points below the black reference curve (i.e., located within the areas marked by gray colors) thus indicate that subsurface material is probed. Interestingly, when a data point exhibits both a smaller peak height and a smaller peak height ratio than a PMMA bulk surface (i.e., is located within the area marked by the dark gray color), we can conlcude that the probed material is below the surface even without reference measurements on thin surface layers. As discussed below for various molecular vibrational bonds, the correlation of peak height ratios and peak heights (Fig. 6b, d) is a rather general and robust method for distinguishing surface and subsurface layers.

We note that for the discrimination of surface and subsurface layers one could apply nano-FTIR peak ratios obtained from other demodulation orders *n* (for example the ratios $\varphi _4^{{\mathrm{max}}}/\varphi _2^{{\mathrm{max}}}$ or$\varphi _3^{{\mathrm{max}}}/\varphi _2^{{\mathrm{max}}}$, see Supplementary Fig. 5), as far as the respective near-field signals are background free and of sufficiently large signal-to-noise ratio.

We apply our findings to clarify the location of PMMA in the SBR/PMMA blend studied in Fig. 1. To that end, we plotted the spectral peak positions $\omega _3^{{\mathrm{max}}}$ and peak height ratios *C* recorded at positions A–D versus the corresponding peak heights $\varphi _3^{{\mathrm{max}}}$ (Fig. 6e, f). We find that the peak position $\omega _3^{{\mathrm{max}}} = 1738\,{\mathrm{cm}}^{ - 1}$ for the largest peak height $\varphi _3^{{\mathrm{max}}}$ (position A) agrees well with the peak position of a thick PMMA surface layer (compare Fig. 6e with Fig. 6a). Together with the large peak height and the sharp material boundaries at the sample surface observed in the AFM mechanical phase image (see description of Fig. 1), we conclude that at position A a thick PMMA surface layer is probed. As discussed above, we use the data point recorded at position A as reference measurement to clarify the location of PMMA at the points B–D. The combined reduction of *C* and $\varphi _3^{{\mathrm{max}}}$ (relative to position A) at each of the positions B–D reliably reveals that a subsurface PMMA layer is probed (as indicated in Fig. 6f by the dark gray area analogous to Fig. 6b, d). This conclusion is supported in Fig. 6e, where we observe strong spectral red shifts down to $\omega _3^{{\mathrm{max}}} = 1732\,{\mathrm{cm}}^{ - 1}$ as the peak height decreases (positions B–D), which is much stronger than that for thin PMMA surface layers (black dots in Fig. 6a and Mastel et. al.^22^).

## Discussion

We have chosen to study the nano-FTIR peak characteristics of the C=O bond, as it represents a typical molecular vibration. Being able to describe this vibration by a Lorenz oscillator model, as most other molecular vibrational bonds, our findings regarding peak shifts and peak height ratios can be assumed to be valid for most molecular vibrational bonds. To corroborate this assumption, we performed a largely extended study. All results of this study are presented in Supplementary Figs. 6–14. In the following, we briefly outline the study and summarize and discuss the main results.

Supplementary Figs. 6–8 present calculations of the momentum-dependent Fresnel reflection coefficient, nano-FTIR peak shifts, and peak height ratios for various C–C–O and C–O–C bonds of surface and subsurface PMMA layers between 1100 and 1300 cm^−1^. The corresponding molecular vibrations differ not only regarding the chemical bonding, but also in oscillator strength, and partially overlap spectrally. Analogous to Supplementary Figs. 6–8, Supplementary Figs. 9, 10 show our results obtained for surface and subsurface polyethylene-oxide layers, which exhibits three C–O stretching modes of different oscillator strengths. The calculations are complemented by experimental results in Supplementary Fig. 11. We also calculated peak shifts and peak height ratios for surface and subsurface layers modeled by a Lorenz oscillator with different high-frequency permittivity (Supplementary Fig. 12). Finally, we study the nano-FTIR peak shifts and peak height ratios of the C=O bond of PMMA subsurface layers as a function of layer depth *d*_2_ and thickness *t*_2_ (Supplementary Fig. 13) and in dependence of the permittivity of the capping layer (Supplementary Fig. 14).

From the results presented in Supplementary Figs. 6–14, we can derive the following general conclusions: (1) nano-FTIR peak positions of subsurface layers are shifted to lower frequencies (red shift) compared with that of surface layers of the same thickness. With increasing depth *d*_2_ of the subsurface layer, the red shift increases. The amount of the red shift, however, strongly depends on the oscillator strength and can vary from being negligibly small (<1 cm^−1^) to several wavenumbers, similar to the C=O peak of PMMA. (2) Most interesting and important, the peak height ratios $C = \varphi _4^{{\mathrm{max}}}/\varphi _3^{{\mathrm{max}}}$ observed for all studied molecular vibrations behave nearly the same as that of the C=O peak, and thus can be considered as a rather robust criterium for distinguishing surface and subsurface layers. (3) With decreasing thickness *t*_2_ of the subsurface layer or increasing permittivity of the capping layer, the peak heights and spectral peak shifts reduce, which in turn reduces the depths *d*_2_ at which a nano-FTIR peak can be practically detected. Generally, and as is the case for any other measurement technique, our results can be applied only for the case that an absorption peak shows sufficient signal-to-noise ratio in the nano-FTIR spectrum and that the peak shifts are large enough to be resolved by typical nano-FTIR instrumentation.

In summary, we found that the peaks in nano-FTIR phase spectra of subsurface organic layers are spectrally red shifted compared with nano-FTIR spectra of the corresponding bulk material, and that the red shift is stronger than the one observed for surface layers when their thickness is reduced^22^. We corroborate our results with a semi-analytical model for calculating nano-FTIR spectra of multilayered samples, which well describes the observed trends. Our model also reveals that peak shifts in nano-FTIR spectra of multilayer samples can be traced back to the sample’s momentum-dependent Fresnel reflection coefficient *β*(*ω*,*q*), provided that chemically induced peak shifts can be excluded. We note that such sample- and momentum determined peak shifts are not an exotic feature of near-field spectroscopy, but also occur in far-field spectroscopy, where the probing momentum is determined by the angle of incidence. We finally demonstrated that surface and subsurface layers can be differentiated by analyzing the ratio of peak heights obtained at different demodulation orders *n*, without the need of theoretical modeling or simulations. Our results will be thus important for the future practical application of nano-FTIR spectroscopy, for example, to distinguish peak shifts caused by sample geometry from peak shifts that are caused by chemical effects such as chemical interaction at material boundaries.

## Methods

### Electromagnetic simulation

The electric near-field intensity distribution |*E*|^2^ in Fig. 1b was simulated with the COMSOL Multiphysics software based on the finite element method. The tip was modelled as a 10 μm-long silicon cone with a 20 nm-thick gold coating and a tip apex radius of 30 nm. The tip was illuminated at an angle of 60° with respect to the surface normal of the sample. The illumination wavelength was *λ* = 5.75 μm, corresponding to *ω* = 1739 cm^−1^). The sample is a 10 nm-thick PS layer (with permittivity *ϵ*_PS_ = 2.5)^46^ on a semi-infinite PMMA substrate (with permittivity *ϵ*_PMMA_ = 1.52 + i0.83)^45^. The distance between tip apex and the sample surface is 2 nm.

### Sample fabrication

For the PMMA/PS test sample, we spin coated a 2% solution of PMMA (molecular weight 495 kDa) dissolved in Anisole at 6000 rpm for 60 s onto a clean silicon substrate and subsequently annealed the sample at 180 °C for 90 s to achieve a smooth PMMA surface. PS was spin coated using the same parameters (but no annealing) on two identical PMMA surfaces as 1.5 and 3% solution in 1-chloropentane (yielding a different range of PS thickness depending on the concentration). We chose 1-chloropentane as chemically selective solvent for PS to ensure a sharp interface between the layers^51^. The wedge shape was obtained by tilting the sample at an angle of ~15° during the PS spin coating step. The heights of the PMMA and PS layers were determined by AFM height measurements, after the underlying silicon substrate was scratched free with freshly cleaned tweezers. For the SBR/PMMA sample, we drop casted a commercially available solution of SBR and PMMA (Nanosurf) onto a silicon substrate.

### Finite dipole model

The employed model parameters are: *A* = 30 nm, *R* = 30 nm, *L* = 200 nm, *g* = 0.65 (*g* describes the amount of induced charge that is relevant for the near-field interaction^15,41^).

## Supplementary information

Supplementary Information

## Data Availability

The data that support the findings of this study are available from the corresponding author upon reasonable request.

## Code availability

The code that supports the findings of this study are available from the corresponding author upon reasonable request.
