# Supplementary material for: Subsurface chemical nanoidentification by nano-FTIR spectroscopy
Source: Nat Commun. 2020 Jul 3;11:3359. doi: 10.1038/s41467-020-17034-6 (PMC7335173; doi:10.1038/s41467-020-17034-6)
Supplement: Supplementary file 1 — Supplementary Information [file 41467_2020_17034_MOESM1_ESM.pdf]

Supplementary Information

Subsurface chemical nanoidentification by nano-FTIR spectroscopy

Mester et al.

Supplementary Methods

Finite Dipole Model

The finite dipole model (FDM)[1,2] is explained and illustrated (Fig. 4) in the main text in a simplified way. With this Supplementary Method section, we provide a more detailed description and illustration (Supplementary Figure 1) of the model. Nano-FTIR signals are described by calculating the scattering coefficient $\sigma = E_{\mathrm{scat}}/E_0$. The tip is approximated as a prolate spheroid with apex radius R and major half-axis length L (here we use $R = 30$ nm and $L = 200$ nm). The electric field E_0 of the external illumination is incident on the tip directly and indirectly via reflection from at the sample surface with the far-field reflection coefficient r. The local electric field at the tip, $E_{\mathrm{loc}} = (1+r)E_0$, induces an electric dipole $p_0 = 2LQ_0$ along the tip-axis.

In the FDM the tip-sample interaction is mediated only via the charge Q_0, which is located at a distance $W_0 = \frac{1.31RL}{L+2R} \approx R$ from the tip apex.[1,2] Q_0 induces an image charge $Q_0' = -\beta Q_0$ in the sample, with $\beta = \frac{\epsilon-1}{\epsilon+1}$ being the quasi-electrostatic Fresnel reflection coefficient for semi-infinite samples. The distance of Q_0' to the sample surface is the same as the height of Q_0 above the sample: $z_0 = H + W_0$ (method of image charges[3]). Q_0' acts back on the tip by inducing the additional charge Q_1 at a distance $W_1 = R/2$ from the tip apex, and its counter charge $-Q_1$ at the spheroid center. Self-consistent treatment of the problem yields

$$Q_1 = \beta Q_0 f_0 + \beta Q_1 f_1, \tag{1}$$

where f_i are geometry factors depending on the tip apex radius R, spheroid major half-axis length L and tip-sample distance H, and given by

$$f_i = \left(g - \frac{R+2H+W_i}{2L}\right) \cdot \frac{\ln\frac{4L}{R+4H+2W_i}}{\ln\frac{4L}{R}} \tag{2}$$

with $i = \{1, 2\}$. The g-factor is a model parameter that describes the amount of induced charge still relevant for the near-field interaction. It is empirically found to be $g \approx 0.7 \pm 0.1$,[2] here we use $g = 0.65$.

The charges $\pm Q_1$ form the electric dipole $p_1 = LQ_1$, yielding for the total electric dipole moment induced in the tip $p = p_0 + p_1$. By using Supplementary Equation (1) and the expressions for p_0 and p_1, we rewrite

$$p \propto E_{\mathrm{loc}}\left(1 + \frac{1}{2}\frac{f_0\beta}{1-f_1\beta}\right), \tag{3}$$

where we have used that Q_0 is proportional to the local electric field at the tip E_{loc}. The effective polarizability of the coupled tip-sample system is defined via $p = \alpha_{\mathrm{eff}} E_{\mathrm{loc}}$ and thus in the FDM it is given by (proportional to)

$$\alpha_{\mathrm{eff}} \propto 1 + \frac{1}{2}\frac{f_0\beta}{1-f_1\beta}. \tag{4}$$

The scattered (far) field of this dipole is measured directly and indirectly via reflection from the sample, yielding $E_{\mathrm{scat}} = (1+r)p$ and thus the tip scattering coefficient

$$\sigma = (1+r)^2\left(1 + \frac{1}{2}\frac{f_0\beta}{1-f_1\beta}\right). \tag{5}$$

Reflected electric monopole field

As explained in the previous Supplementary Methods section, the FDM describes the tip-sample interaction in a simple image charge model: The tip produces an electric field distribution similar to that of a charge Q_0, which induces a mirror charge Q_0' in the sample (i.e. for bulk samples $Q_0' = -\beta Q_0$), which then acts back onto the tip (Supplementary Equation (1)).

In order to derive an effective $\bar{\beta}$, such that the image charge of Q_0 in multilayered samples is described by $Q_0' = -\bar{\beta} Q_0$, we analyse and compare the electric field distributions produced by Q_0 and Q_0', the latter corresponding to the field of Q_0 after reflection at the sample surface (method of image charges[3]).

We express in the following (i) the electric field $\mathbf{E}$ of the monopole Q_a (in the absence of a sample) in the angular spectrum representation[4] and (ii) the electric field reflected from the multilayered sample, $\mathbf{E}_{\mathrm{refl}}$, from which we derive (iii) the effective (momentum-integrated) near-field reflection coefficient $\bar{\beta}$ that is used in the main text. Such treatment fully accounts for the evanescent part of the plane wave spectrum necessary for proper description of the near-field tip-sample interaction.

(i) Electric monopole field

The electric field of a point charge $Q_{\mathrm{a}} \mathrm{e}^{-\mathrm{i}\omega t}$ (located in the origin of a coordinate system) is given at an arbitrary point $\mathbf{r} = (x, y, z)$ in space by[5]

$$\mathbf{E}(\mathbf{r}) = -\boldsymbol{\nabla}\Phi = -\frac{Q_{\mathrm{a}}}{4\pi\epsilon_0}\boldsymbol{\nabla}\frac{\mathrm{e}^{\mathrm{i}\mathbf{kr}}}{r}, \quad (6)$$

where Φ is the electric potential of the point charge, the nabla operator is given by $\boldsymbol{\nabla} = (\frac{\partial}{\partial x}, \frac{\partial}{\partial y}, \frac{\partial}{\partial z})$ and the oscillation frequency ω is related to the electromagnetic wave momentum $k = \omega/c$. We obtain the angular spectrum representation of Supplementary Equation (6) by using the Weyl identity,[4]

$$\frac{\mathrm{e}^{\mathrm{i}\mathbf{kr}}}{r} = \frac{\mathrm{i}}{2\pi}\iint_{-\infty}^{\infty}\frac{\mathrm{e}^{\mathrm{i}k_x x+\mathrm{i}k_y y+\mathrm{i}k_z z}}{k_z}dk_x dk_y, \quad (7)$$

yielding for the electric field of the monopole field

$$\mathbf{E}(\mathbf{r}) = -\frac{Q_{\mathrm{a}}}{4\pi\epsilon_0}\boldsymbol{\nabla}\frac{\mathrm{i}}{2\pi}\iint_{-\infty}^{\infty}\frac{\mathrm{e}^{\mathrm{i}k_x x+\mathrm{i}k_y y+\mathrm{i}k_z z}}{k_z}\,dk_x dk_y$$

$$= \frac{Q_{\mathrm{a}}}{8\pi^2\epsilon_0}\iint_{-\infty}^{\infty}\frac{\mathrm{e}^{\mathrm{i}k_x x+\mathrm{i}k_y y+\mathrm{i}k_z z}}{k_z}\begin{pmatrix}k_x\\k_y\\k_z\end{pmatrix}dk_x dk_y. \quad (8)$$

For simplicity, we restrict ourselves to analysing the z-component E_z of the electric field along the z-axis ($x = 0, y = 0$), which we justify by the elongated shape of the probing tip (providing near fields below the tip apex that are essentially polarized along the z-direction) and the rotational symmetry of the problem:

$$E_z(0,0,z) = \frac{Q_{\mathrm{a}}}{8\pi^2\epsilon_0}\iint_{-\infty}^{\infty}\frac{\mathrm{e}^{\mathrm{i}k_z z}}{k_z}k_z\,dk_x dk_y. \quad (9)$$

The FDM models the nano-FTIR probing tip as a prolate spheroid of length $2L$, which is much shorter than the wavelength of infrared radiation used in our experiments. Thus, we further simplify Supplementary Equation (9) by taking the electrostatic limit (which leads to the condition $k_z = \mathrm{i}\sqrt{k_x^2 + k_y^2}$):

$$E_z(z) = \frac{Q_\mathrm{a}}{8\pi^2\epsilon_0} \iint_{-\infty}^{\infty} \mathrm{e}^{-(k_x^2+k_y^2)z}\, dk_x dk_y. \quad (10)$$

We obtain our final expression for $E_z(z)$ after coordinate transformation from cartesian coordinates (k_x, k_y, k_z) to cylindrical coordinates $(q = \sqrt{k_x^2 + k_y^2}, k_\varphi, k_z)$ and integration over k_φ:

$$E_z(z) = \frac{Q_\mathrm{a}}{4\pi\epsilon_0} \int_0^{\infty} q\mathrm{e}^{-qz}\, dq. \quad (11)$$

As consistency check, we perform the integration over q and reproduce the typical z^{-2} dependence for the electric monopole field:

$$E_z(z) = \frac{Q_\mathrm{a}}{4\pi\epsilon_0} \frac{1}{z^2} \quad (12)$$

(ii) Reflected electric monopole field

We now derive the field after reflection from the (multilayer) sample surface, starting from Supplementary Equation (11). The monopole field is purely p-polarized (due to the rotational symmetry of the problem), which allows us to express all reflections at the sample surface by the quasi-electrostatic Fresnel reflection coefficient for p-polarized light $\beta(q)$ which is given in Equation (5) of the main text. As illustrated in Fig. 4b (red dashed arrow), the electric monopole field extends from the position of the monopole to the sample surface (yielding the term $\exp(-qz_\mathrm{a})$ with $z_\mathrm{a} = H + a$), where it is reflected via $\beta(q)$ and extends back to arbitrary z (yielding $\exp(-qz)$). The reflected electric field of the monopole Q_a at the position z (measured from the sample surface) is thus given by

$$E_{z,\mathrm{refl}}(z) = \frac{Q_\mathrm{a}}{4\pi\epsilon_0} \int_0^{\infty} q\mathrm{e}^{-qz_\mathrm{a}}\beta(q)\mathrm{e}^{-qz}\, dq. \quad (13)$$

(iii) Reflection coefficient $E_{z,\mathrm{refl}}(z)/E_z(z)$

In order to obtain the momentum-integrated near-field reflection coefficient for multilayer samples, we calculate the ratio $E_{z,\mathrm{refl}}(z)/E_z(z')$, where $E_z(z')$ is the electric field distribution produced by an effective point charge Q_a and $E_{z,\mathrm{refl}}(z)$ is the electric field distribution produced by Q_a that is reflected at the multi-layered sample surface. We distinguish z and z' in order to remind that the origin of the nominator ($z = 0$) lies in the sample surface plane (Supplementary Equation (13)), while in the denominator the origin ($z' = 0$) lies in the monopole Q_a (which is located at a height $z_\mathrm{a} = H + a$ above the sample surface plane, Supplementary Equation (11)). By using Supplementary Equations (11) and (13) we obtain

$$\frac{E_{z,\mathrm{refl}}(z)}{E_z(z')} = \frac{\int_0^{\infty} \beta(q) q\mathrm{e}^{-qz_\mathrm{a}}\mathrm{e}^{-qz} dq}{\int_0^{\infty} q\mathrm{e}^{-q\,z'} dq}. \quad (14)$$

Note that Supplementary Equation (14) is independent of the momentum q (after integration).

In principle, Supplementary Equation (14) can be evaluated at arbitrary z, however, in order to describe the tip-sample interaction, we make an approach similar to Aizpurua et. al.[6] and Fei et. al.[7] and evaluate the reflected field at the position of the charge Q_a itself, $E_{z,\mathrm{refl}}(z = z_\mathrm{a})$, which we compare with the (incident) monopole field $E_z(z' = 2z_\mathrm{a})$, ensuring that we evaluate the incident and reflected field at the same distance from the respective charges (mirror charge in case of the reflected field). We thus define the effective near-field reflection coefficient (which is valid at the position of Q_a) as

$$\frac{E_{z,\mathrm{refl}}(z=z_\mathrm{a})}{E_z(z'=2z_\mathrm{a})} = \frac{\int_0^\infty \beta(q) q \mathrm{e}^{-2qz_\mathrm{a}} dq}{\int_0^\infty q \mathrm{e}^{-2qz_\mathrm{a}} dq} =: \bar{\beta} \qquad (15)$$

We note that the integral in the nominator of Supplementary Equation (15) contains a coupling weight function (Equation (7) in the main text) that is proportional to q, similar to expressions found in the work of Hauer et. al.[8] On the other hand, a q^2-dependency is found in similar momentum-integrals contained in the point dipole model for multi-layered samples[6] and the lightning rod model[9,10], as they are derived from a reflected dipole field, rather than a reflected monopole field.

Supplementary Figures

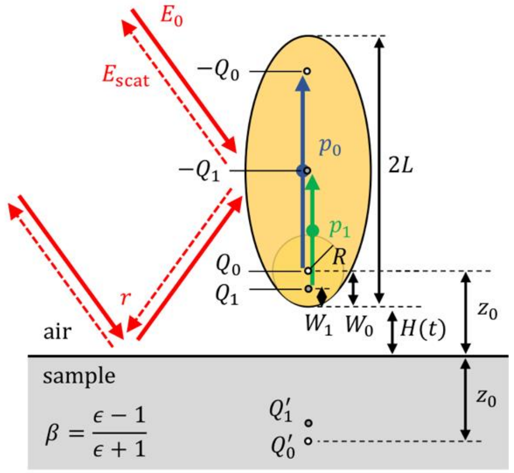

Supplementary Figure 1: Detailed illustration of the finite dipole model for bulk samples. The nano-FTIR tip is modelled as a prolate spheroid of length $2L$ and apex radius R, which is located at height $H(t)$ above a bulk sample with permittivity ϵ and electrostatic reflection coefficient $\beta = (\epsilon - 1)/(\epsilon + 1)$. The incident electric field E_0 induces the primary electric dipole p_0, which consists of the point charges $\pm Q_0$ which are located at distances W_0 from the tip apexes. The point charge Q_0 creates a mirror charge Q_0' in the sample, which yields an additional (near-field induced) dipole p_1 in the tip. The dipole p_1 consists of the point charges Q_1 at a distance W_1 from the sample-near tip apex and $-Q_1$ in the spheroid center. The model accounts for far-field illumination and detection of the tip-scattered field E_{scat} via reflection at the sample surface, with Fresnel reflection coefficient r (indicated by red arrows).

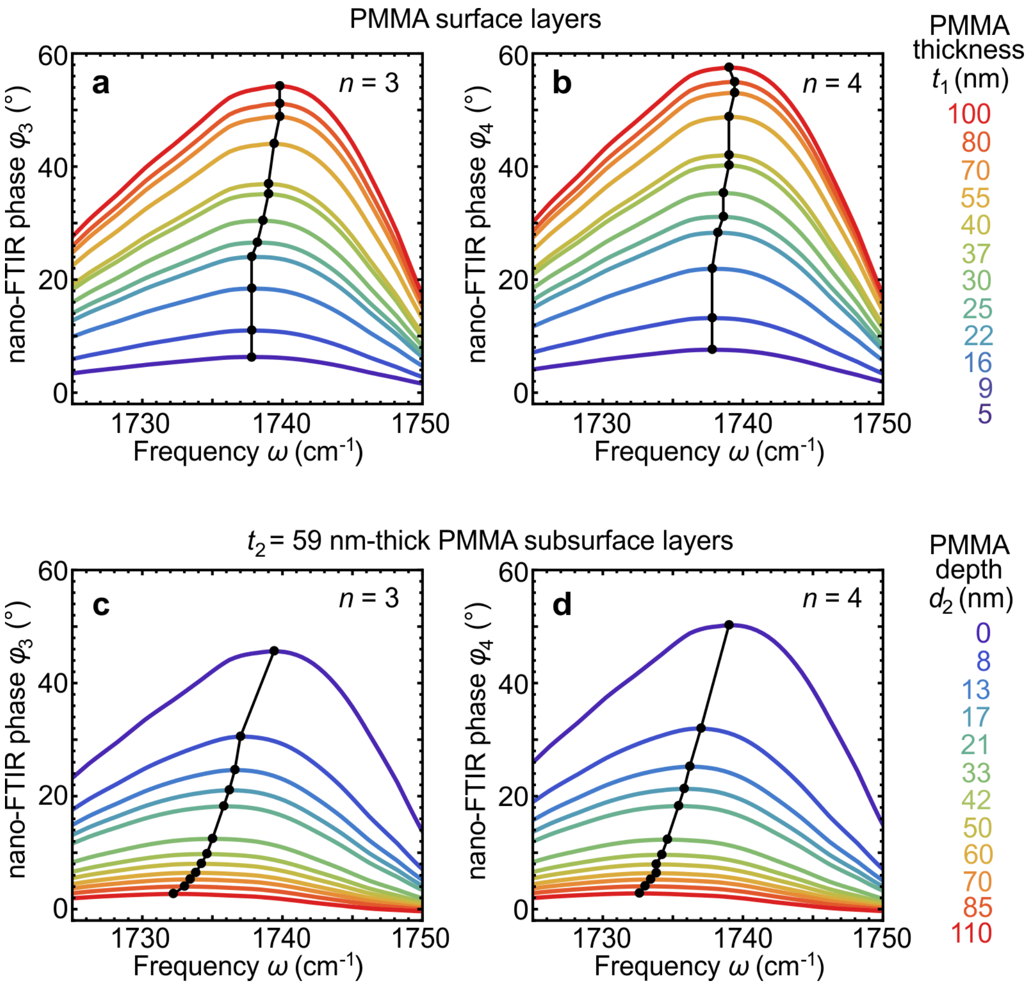

Supplementary Figure 2: Calculated nano-FTIR phase spectra of (a,b) thin PMMA layers of varying thickness t_1 on silicon and of (c,d) a $t_2 = 59$ nm-thin PMMA layer silicon buried at different depths below PS, calculated with a spectral resolution of $\Delta\omega = 0.4\ \mathrm{cm}^{-1}$. As in the experiment, the spectral baseline (non-absorbing frequency range; in calculated spectra around $\omega = 1770$ to $1780\ \mathrm{cm}^{-1}$) has been subtracted before finding the maxima, defined as points with the highest φ_n. The black dots indicate the calculated maxima used in Fig. 3 of the main text.

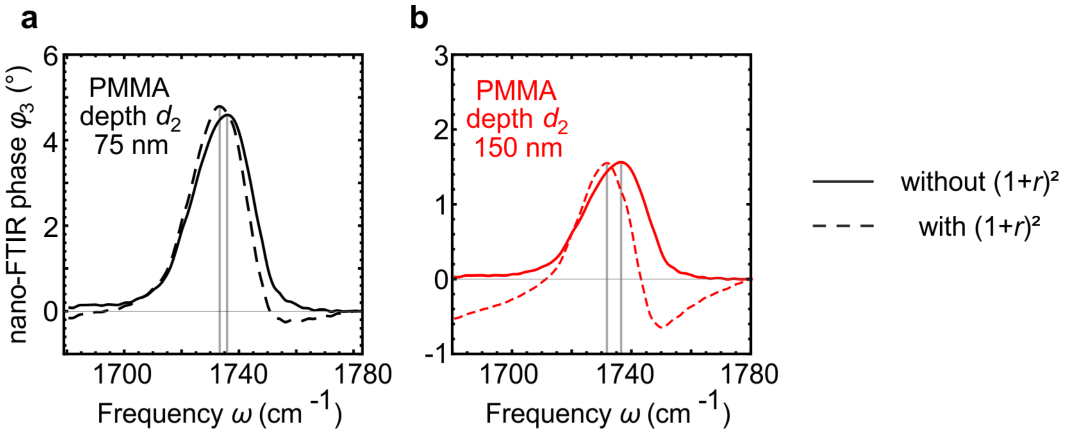

Supplementary Figure 3: Calculated nano-FTIR phase spectra with and without far-field reflection at sample. Calculated nano-FTIR phase spectra of a t_2 = 59 nm-thin subsurface PMMA layer on a silicon substrate, located at depth (a) d_2 = 75 nm and (b) d_2 = 150 nm below PS. Dashed and solid lines are calculated with and without the factor $(1+r)^2$ in Equation (1) of the main text, which accounts for far-field illumination and detection via the sample surface. Vertical gray lines indicate peak positions. The figure shows that the factor $(1+r)^2$ yields an additional red-shift of the peak (panel a) and an increasingly pronounced dispersive line shape with increasing depth of the subsurface layer. Note that in experiment this effect appears already at smaller depth d_2.

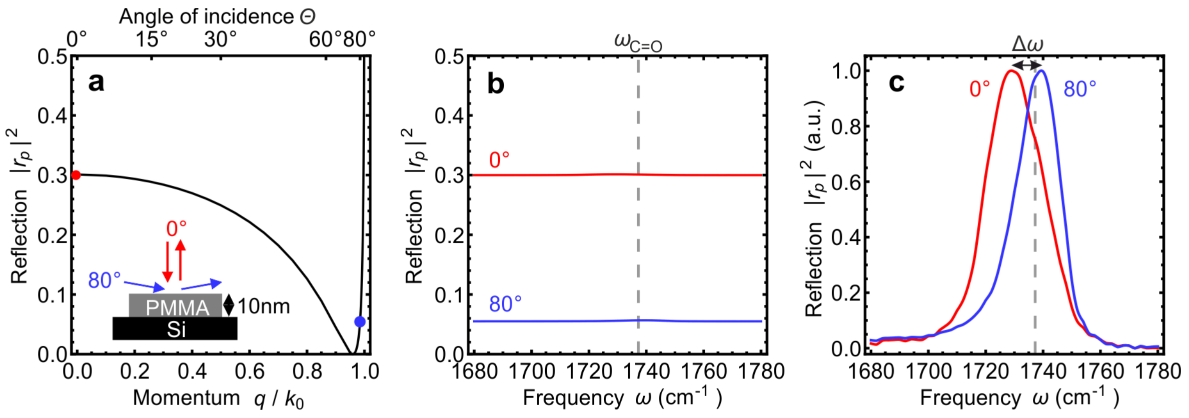

Supplementary Figure 4: Momentum-dependent spectral peak shifts in far-field infrared reflection spectroscopy. Calculated reflected power $|r_p|^2$ of p-polarized infrared far-field radiation, reflected from the surface of a 10 nm-thin PMMA layer on a silicon substrate (as illustrated in the inset), for (a) varying in-plane momenta q which correspond to varying angles of incidence Θ via $q = k_0 \cdot \sin(\Theta)$ for a fixed frequency $\omega = 1737\ \mathrm{cm}^{-1}$ near the $\mathrm{C=O}$ vibrational mode of PMMA, (b) varying frequency for normal-incidence (0°, red) and grazing-incidence (80°, blue). (c) Same as panel b, but shown after baseline subtraction and normalization for better visibility of the angle-dependent spectral peak-shift $\Delta\omega \approx 10\ \mathrm{cm}^{-1}$.

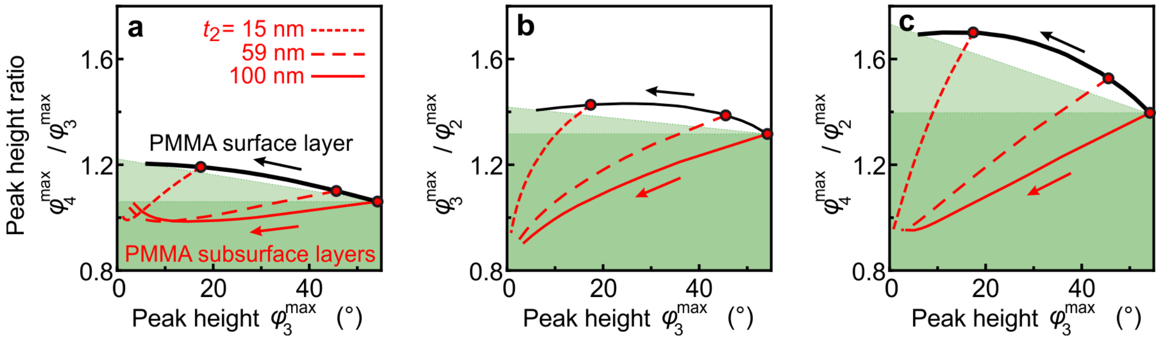

Supplementary Figure 5: Comparison of calculated peak height ratios for different signal demodulation orders. (a) $\varphi_4^{\max}/\varphi_3^{\max}$, (b) $\varphi_3^{\max}/\varphi_2^{\max}$ and (c) $\varphi_4^{\max}/\varphi_2^{\max}$ of PMMA surface (black curve) and PMMA subsurface (red curves) layers are plotted versus the corresponding peak heights $\varphi_3^{\max}$. Arrows indicate decreasing PMMA surface layer thickness t_1 (black) and increasing PMMA subsurface layer depth d_2 (red). Subsurface PMMA layer thicknesses are t_2 = 15 nm (dotted red line), t_2 = 59 nm (dashed red line) and t_2 = 100 nm (solid red line). (a-c) Green areas indicate the data spaces that correspond to subsurface material. The figure shows that peak height ratios obtained from various harmonics can be used to distinguish whether and infrared absorbing layer is at the surface or below the surface.

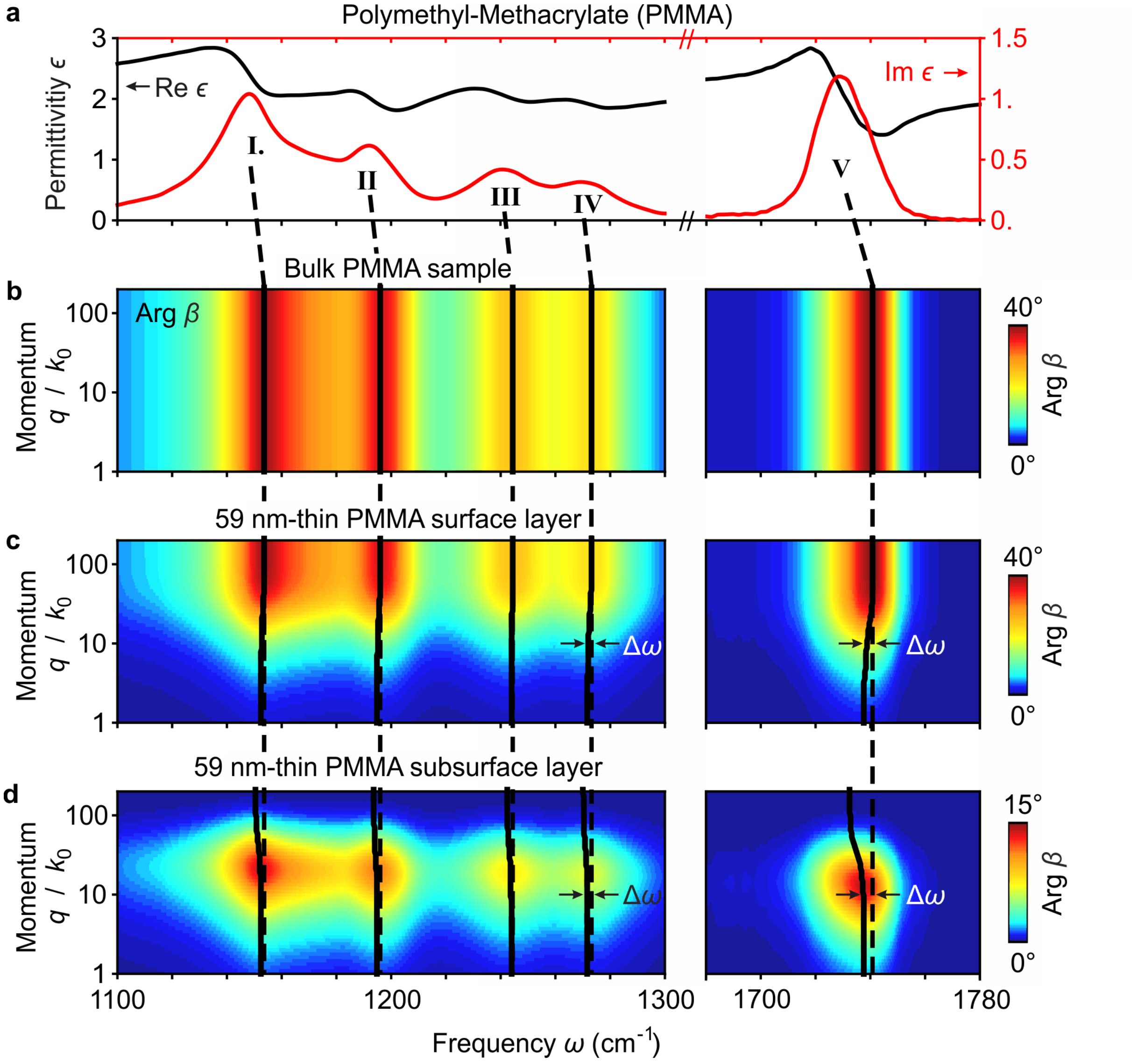

Supplementary Figure 6: Momentum-dependent spectral peak shifts in Fresnel reflection coefficient for various (partially spectrally overlapping) vibrational modes of PMMA. (a) Real (black) and imaginary (red) parts of the dielectric function of PMMA. Peaks in Im[ϵ] correspond to the asymmetric C-O-C (marked I and II), the symmetric C-C-O (marked III and IV) and the C=O (marked V) vibrational stretching modes respectively. (b-d) Phase of the quasi-electrostatic Fresnel reflection coefficient (Arg β) as a function of frequency ω and momentum q for (b) bulk PMMA, (c) a t_1 = 59 nm-thin surface PMMA layer and (d) a t_2 = 59 nm-thin subsurface PMMA layer located at depth d_2 = 20 nm below PS. Both layers in (c,d) are placed on a silicon substrate. The continuous black lines indicate the frequencies where Arg β has its maxima. The dashed vertical lines mark the maxima of bulk PMMA and act as reference frequencies for determining the peak-shifts $\Delta\omega$. The figure shows that: (*1*) all peaks of thin PMMA surface layers shift to lower frequencies (red-shift) with decreasing momenta. (*2*) all peaks of PMMA subsurface layers red-shift with increasing momenta. (*3*) The red-shift is stronger for subsurface layers as compared to surface layers. (*4*) Importantly, the peak shifts occur for weaker and partially spectrally overlapping peaks (I-IV), as well as for the isolated and strong peak (V). Zoom-ins of the panels (c,d) are provided in Supplementary Figure 7.

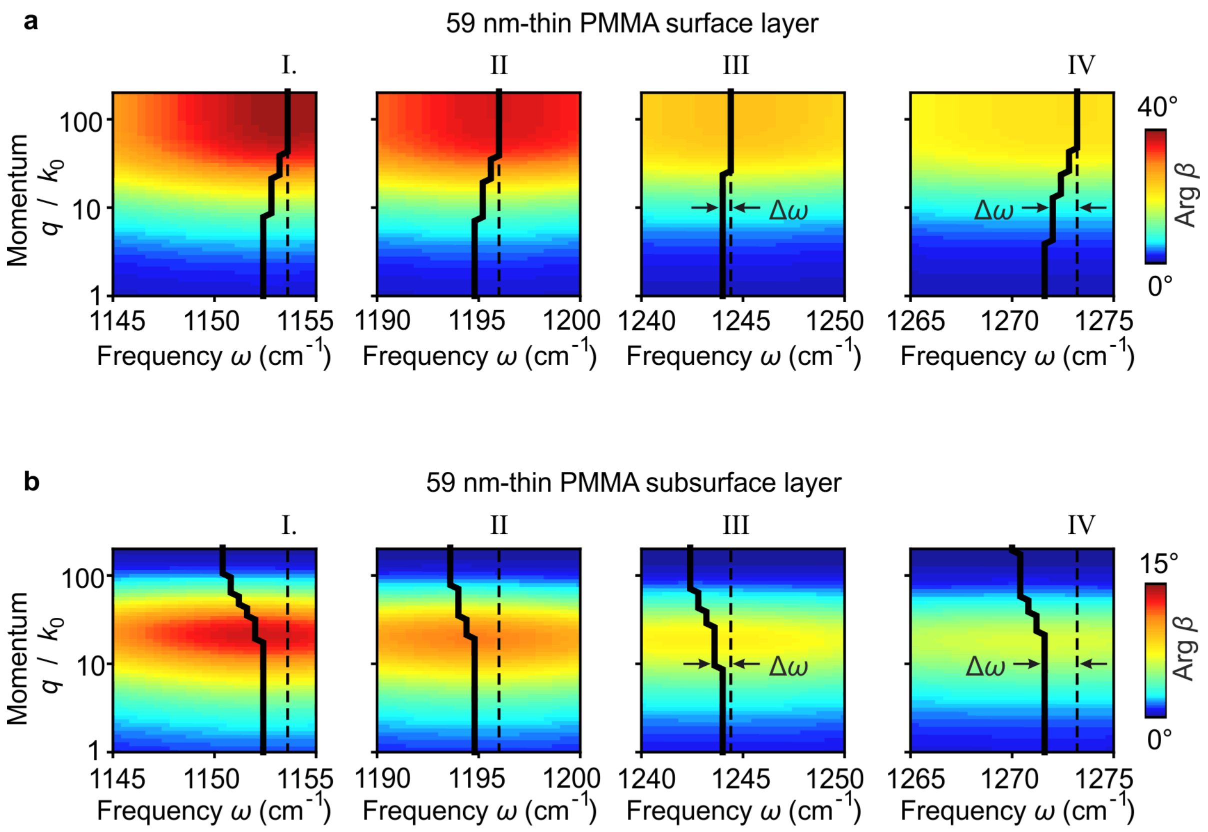

Supplementary Figure 7: Zoom-in to Supplementary Figure 6c,d.

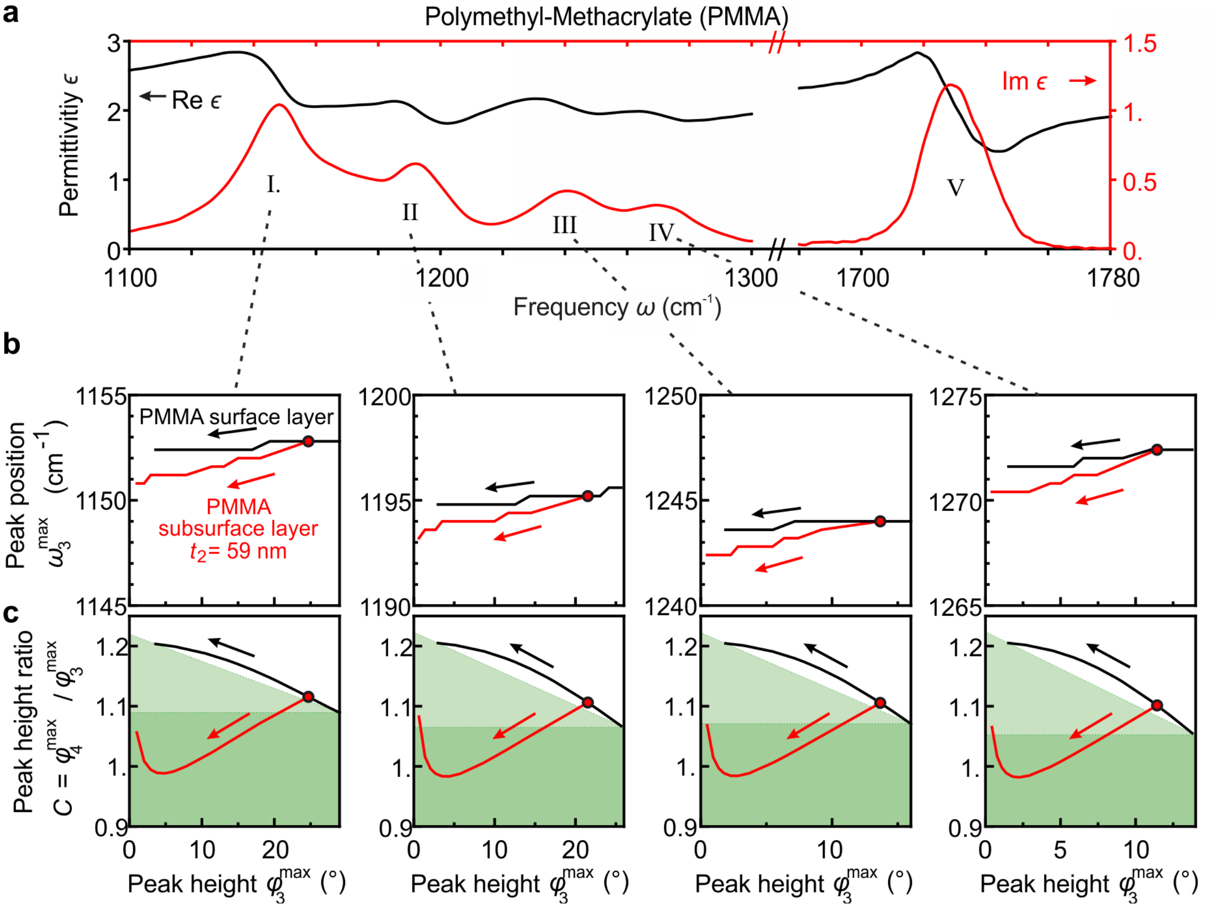

Supplementary Figure 8: Correlation of nano-FTIR peak characteristics for various (partially spectrally overlapping) vibrational modes of PMMA. (a) Real (black) and imaginary (red) parts of the dielectric function of PMMA. Peaks in Im[ϵ] correspond to the asymmetric C-O-C (marked I and II), the symmetric C-C-O (marked III and IV) and the C=O (marked V) vibrational stretching modes respectively. (b) Calculated spectral peak positions $\omega_3^{\max}$ and (c) peak height ratios C of PMMA surface (black symbols) and PMMA subsurface (red symbols) layers are plotted versus the corresponding peak heights $\varphi_3^{\max}$, for the vibrational modes I-IV of PMMA. Arrows indicate decreasing PMMA surface layer thickness t_1 (black) and increasing PMMA subsurface layer depth d_2 (red). Subsurface PMMA layer thickness is t_2 = 59 nm. Green areas in (c) indicate the data spaces that correspond to subsurface material. The figure shows for various (partially spectrally overlapping) vibrational modes of PMMA that: (*1*) nano-FTIR peak positions shift to lower frequencies (red-shift) when the thickness t_1 of a surface layer decreases or when the depth d_2 of a subsurface layer increases. (*2*) The red-shift is stronger for subsurface layers as compared to surface layers. (*3*) Most interesting and important, the peak height ratios $C = \varphi_4^{\max}/\varphi_3^{\max}$ observed for all molecular vibrations of PMMA behave nearly the same as that of the C=O peak (Fig. 6), and thus can be considered as a rather robust criterium for distinguishing surface and subsurface layers.

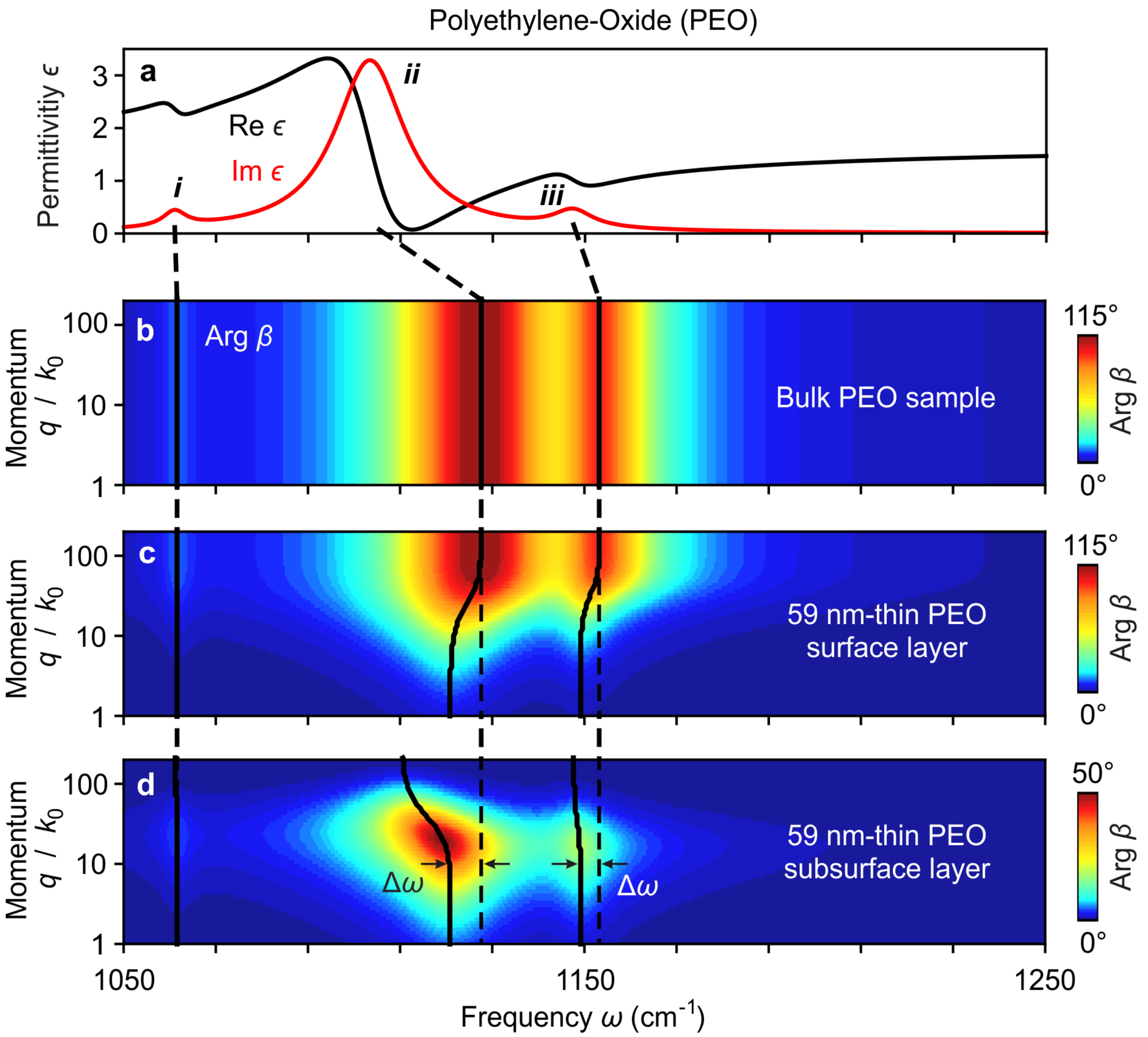

Supplementary Figure 9: Momentum-dependent spectral peak shifts in Fresnel reflection coefficient for differently strong vibrational modes of Polyethylene-oxide (PEO). (a) Real (black) and imaginary (red) parts of the dielectric function of PEO. Peaks in Im[ϵ] (marked *i*-*iii*) correspond to C-O stretching modes. (b-d) Phase of the quasi-electrostatic Fresnel reflection coefficient (Arg β) as a function of frequency ω and momentum q for (b) bulk PEO, (c) a t_1 = 59 nm-thin surface PEO layer and (d) a t_2 = 59 nm-thin subsurface PEO layer located at depth d_2 = 20 nm below PS. Both layers in (c,d) are placed on a silicon substrate. The continuous black lines indicate the frequencies where Arg β has its maxima. The dashed vertical lines mark the maxima of bulk PEO and act as reference frequencies for determining the peak-shifts $\Delta\omega$. The figure shows (for other peaks than of PMMA) that: (*1*) peaks of thin PEO surface layers shift to lower frequencies (red-shift) with decreasing momenta. (*2*) peaks of PEO subsurface layers red-shift with increasing momenta. (*3*) The red-shift is stronger for subsurface layers as compared to surface layers. (*4*) The magnitude of $\Delta\omega$ largely varies from peak to peak and is not directly related to the strength of a vibrational mode; i.e. the bonds *i* and *iii* are similar in Im[ϵ] but different in $\Delta\omega$, on the other hand, the bonds *ii* and *iii* are similar in $\Delta\omega$ but different in Im[ϵ].

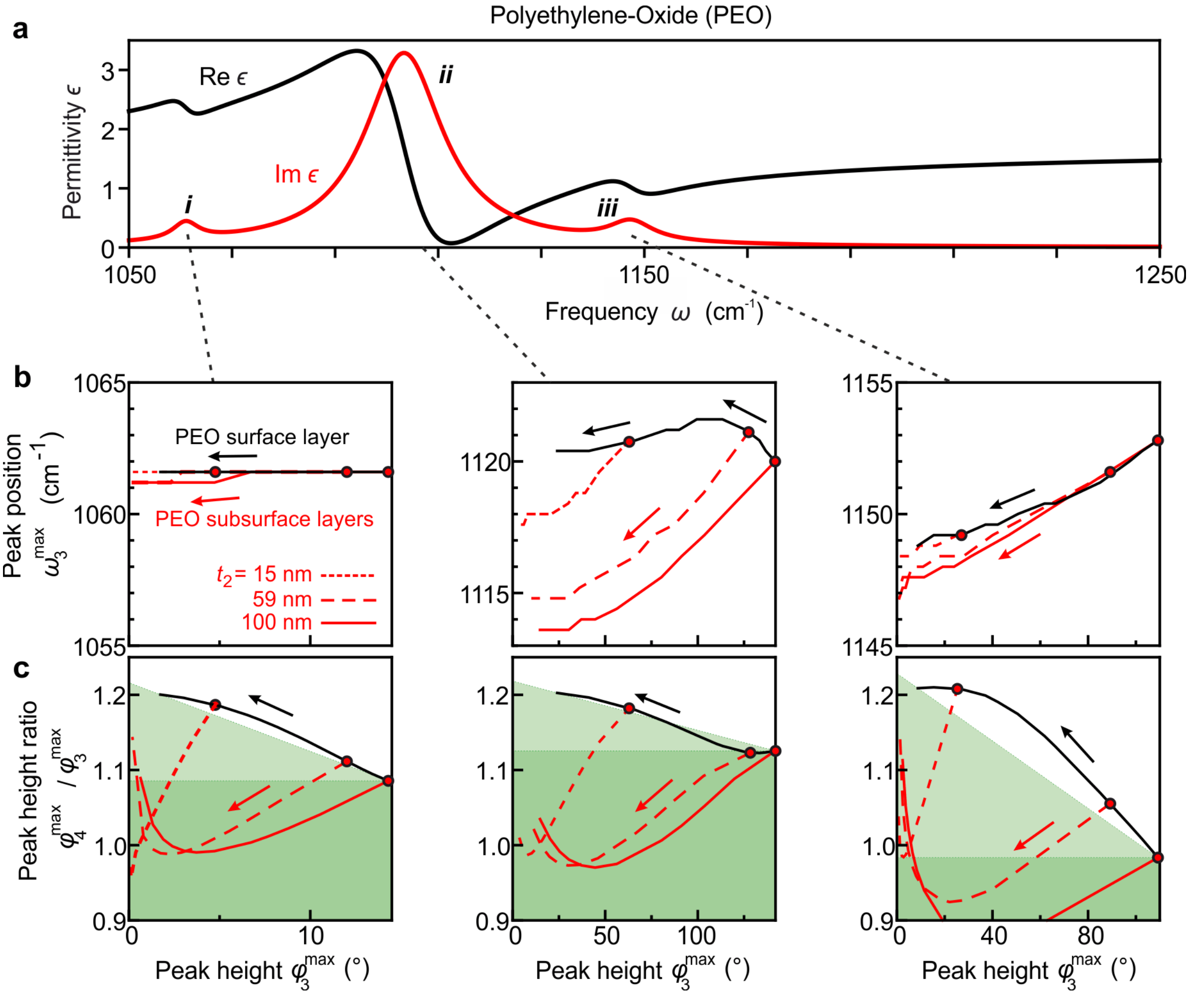

Supplementary Figure 10: Correlation of nano-FTIR peak characteristics for various (differently strong) vibrational modes of PEO. (a) Real (black) and imaginary (red) parts of the dielectric function of PEO. Peaks in Im[ϵ] (marked *i-iii*) correspond to C-O vibrational stretching modes. (b) Calculated spectral peak positions $\omega_3^{\max}$ and (c) peak height ratios C of PEO surface (black symbols) and PEO subsurface (red symbols) layers are plotted versus the corresponding peak heights $\varphi_3^{\max}$, for the vibrational modes *i-iii* of PEO. Arrows indicate decreasing PEO surface layer thickness t_1 (black) and increasing PEO subsurface layer depth d_2 (red). Subsurface PEO layer thicknesses are t_2 = 15 nm (dotted red line), t_2 = 59 nm (dashed red line) and t_2 = 100 nm (solid red line). Green areas in (c) indicate the data spaces that correspond to subsurface material. The figure shows for differently strong vibrational modes of PEO that: (*1*) nano-FTIR peak positions shift to lower frequencies (red-shift) when the thickness t_1 of a surface layer decreases or when the depth d_2 of a subsurface layer increases. (*2*) The red-shift is stronger for subsurface layers as compared to surface layers. (*3*) Most interesting and important, the peak height ratios $C = \varphi_4^{\max}/\varphi_3^{\max}$ observed for all molecular vibrations of PEO behave nearly the same as that of the C=O peak of PMMA (Fig. 6), and thus can be considered as a rather robust criterium for distinguishing surface and subsurface layers.

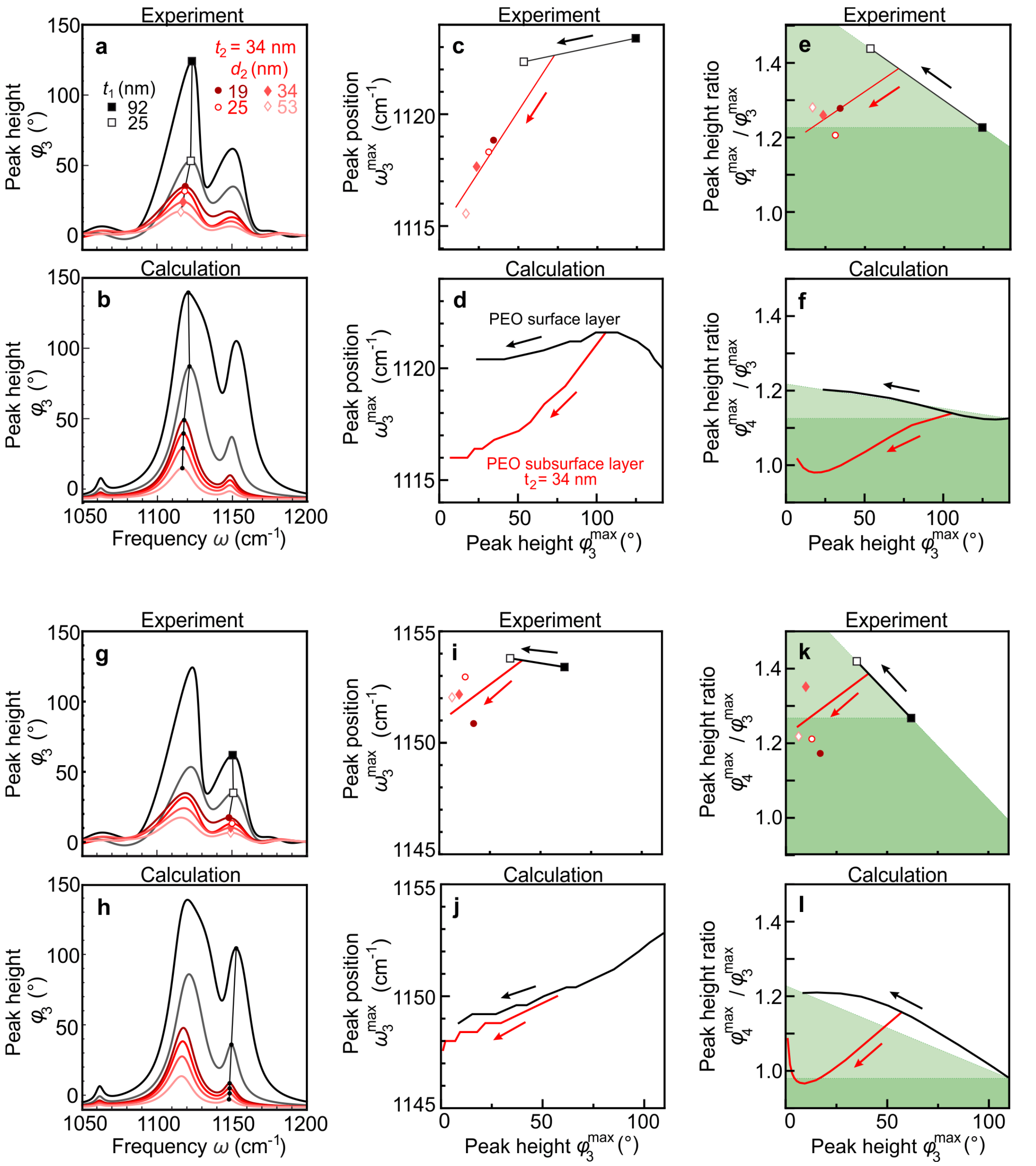

Supplementary Figure 11: Comparison of experimental and calculated nano-FTIR peak characteristics for PEO. (a) Experimental and (b) calculated nano-FTIR phase spectra of PEO surface layers with thickness t_1 = { 25, 92 } nm (black symbols) and PEO subsurface layers with average thickness t_2 = 34 nm and depth d_2 = { 19, 25, 34, 53 } nm (red symbols). (c) Spectral peak positions ω_3^{max} and (e) peak height ratios C for the peak centered at 1115 cm^{-1} of PEO surface (black symbols) and PEO subsurface (red symbols) layers are plotted versus the corresponding peak heights $\varphi_3^{\max}$. Arrows indicate decreasing PEO surface layer thickness t_1 (black) and increasing PEO subsurface layer depth d_2 (red). (d,f) Calculation results analogous to Figures 6c and e. Additionally, results for a large range of PEO surface layer thicknesses t_1 and PEO subsurface layer depths d_2 are shown. (e,f) Green areas indicate the data spaces that correspond to subsurface material. (g-l) Data analogous to panels a-f, for the peak centered at 1150 cm^{-1}. Spectral resolution of the experiments 17 cm^{-1}. The figure shows that (*1*) experimental and calculated nano-FTIR peak positions of subsurface PEO layers shift to lower frequencies (red-shift) compared to that of surface PEO layers of similar thickness. (*2*) The amount of the red-shift varies, depending on which peak is analysed. (*3*) Most interesting and important, the peak height ratios $C = \varphi_4^{\max}/\varphi_3^{\max}$ observed for all PEO subsurface layers are smaller than those of PEO surface layers, corroborating their use as a rather robust criterium for distinguishing surface and subsurface layers.

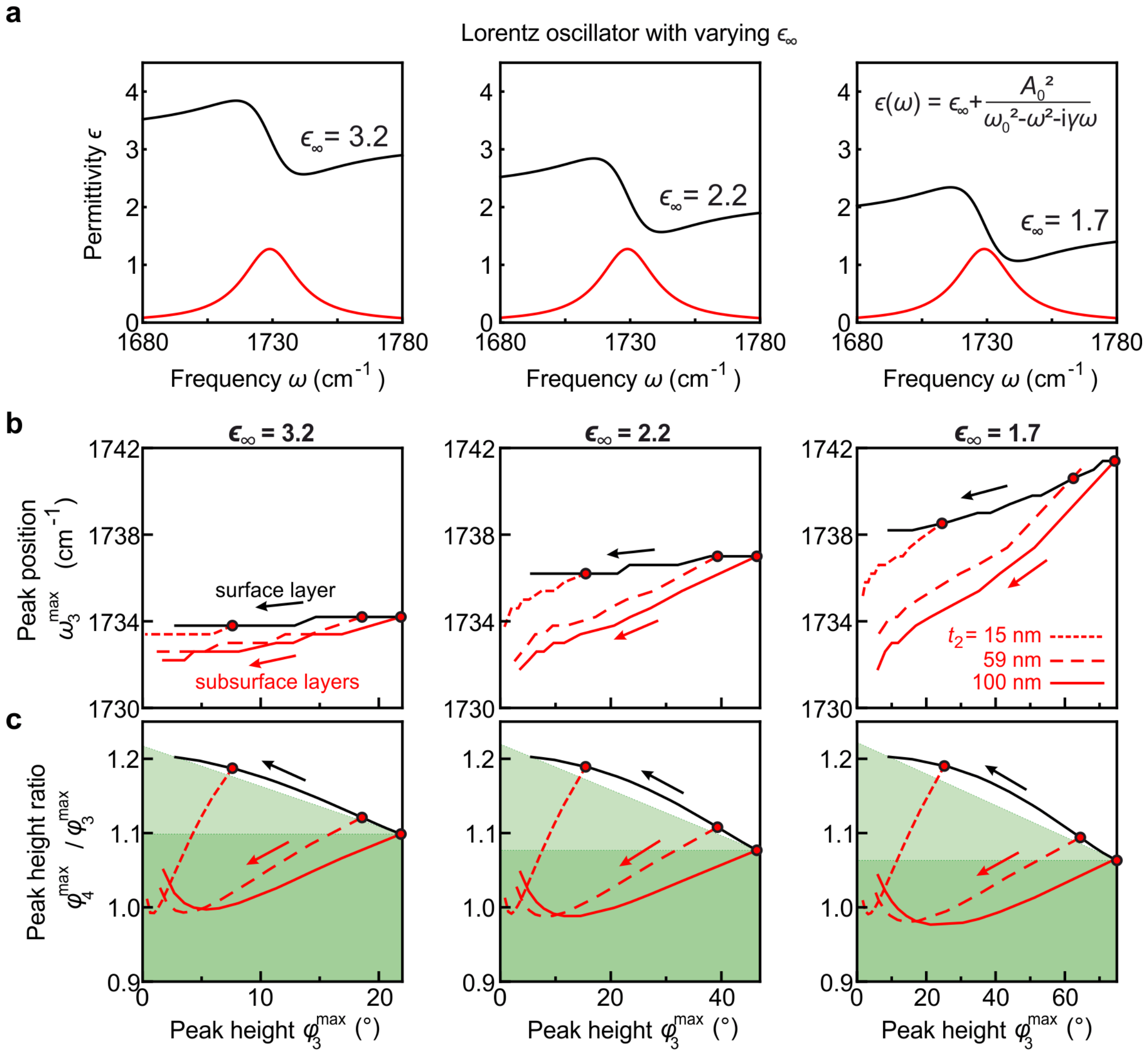

Supplementary Figure 12: Correlation of nano-FTIR peak characteristics for a model Lorentz oscillator with varying high-frequency permittivities. (a) Real (black) and imaginary (red) parts of dielectric functions modelled by Lorentz oscillators with different high-frequency permittivities ϵ_∞ = { 3.2, 2.2, 1.7 } and fixed ω_0 = 1739 cm^{-1}, γ = 26 cm^{-1}, A_0 = 240 cm^{-1}. (b) Calculated spectral peak positions $\omega_3^{\max}$ and (c) peak height ratios C of surface (black symbols) and subsurface (red symbols) layers are plotted versus the corresponding peak heights $\varphi_3^{\max}$, for layers with the permittivities shown in panel a. Arrows indicate decreasing surface layer thickness t_1 (black) and increasing subsurface layer depth d_2 (red). Subsurface layer thicknesses are t_2 = 15 nm (dotted red line), t_2 = 59 nm (dashed red line) and t_2 = 100 nm (solid red line). Green areas in (c) indicate the data spaces that correspond to subsurface material. The figure shows for vibrational modes modelled by Lorentz oscillators with different high-frequency permittivities ϵ_∞ that: (*1*) nano-FTIR peak positions shift to lower frequencies (red-shift) when the thickness t_1 of a surface layer decreases or when the depth d_2 of a subsurface layer increases. (*2*) The red-shift is stronger for subsurface layers as compared to surface layers. (*3*) Most interesting and important, the peak height ratios $C = \varphi_4^{\max}/\varphi_3^{\max}$ observed for all modelled vibrations behave nearly the same as that of the C=O peak of PMMA (Fig. 6), and thus can be considered as a rather robust criterium for distinguishing surface and subsurface layers.

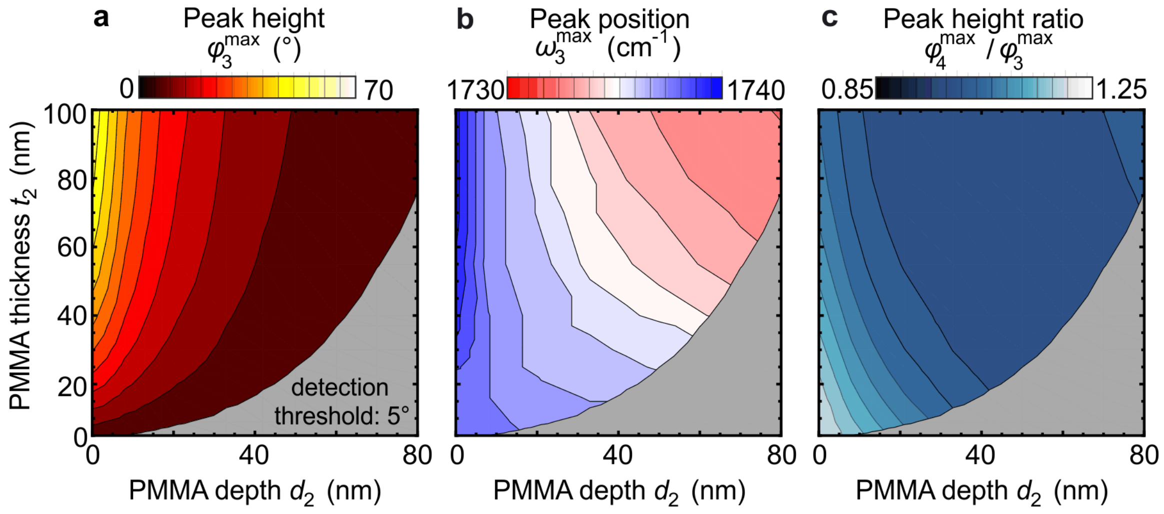

Supplementary Figure 13: Nano-FTIR peak characteristics as function of layer depth and thickness. (a) Peak heights $\varphi_3^{\max}$, (b) spectral peak positions $\omega_3^{\max}$ and (c) peak height ratios C of PMMA subsurface layers are plotted as a function of PMMA depth d_2 and PMMA thickness t_2. Gray areas indicate peak heights $\varphi_3^{\max} < 5°$, which we consider to be below the detection threshold. The figure shows that (*1*) for each PMMA layer thickness t_2 the nano-FTIR peak positions shift to lower frequencies (red-shift) when the depth d_2 of a subsurface layer increases. (*2*) The red-shift is stronger for PMMA subsurface layers with larger thickness t_2. (*3*) With decreasing thickness t_2 of the subsurface layer the peak heights reduce, which in turn reduces the depths d_2 at which a nano-FTIR peak can be practically detected. (*4*) Most interesting and important, the peak height ratios $C = \varphi_4^{\max}/\varphi_3^{\max}$ observed for all thicknesses t_2 decreases as the depth d_2 increases, corroborating the robustness as criterium for distinguishing surface and subsurface layers.

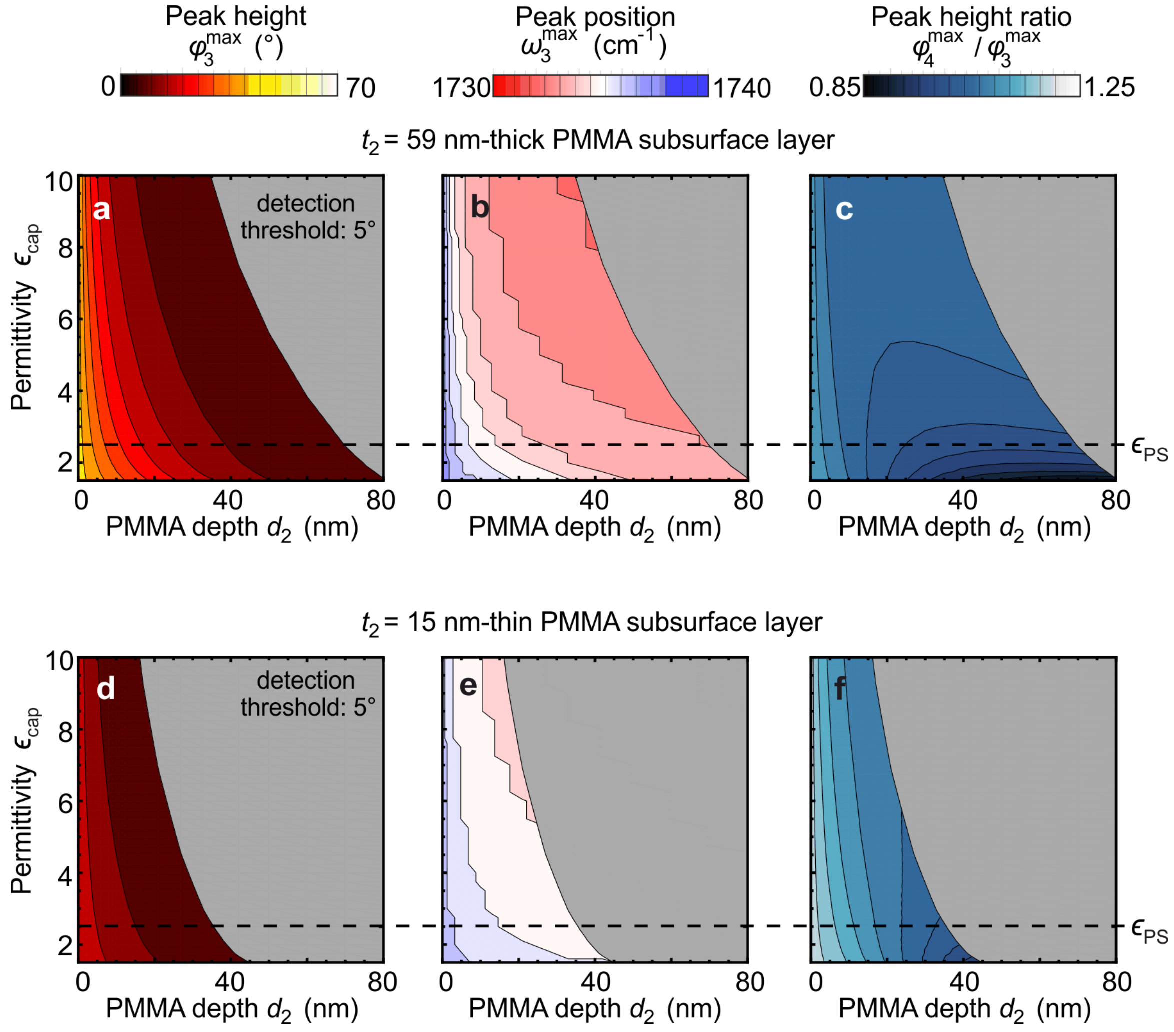

Supplementary Figure 14: Nano-FTIR peak characteristics as function of capping layer permittivity and thickness. (a) Peak heights $\varphi_4^{\max}$, (b) spectral peak positions $\omega_4^{\max}$ and (c) peak height ratios C of a t_2 = 59 nm-thick PMMA subsurface layer are plotted as a function of PMMA depth d_2 and capping layer permittivity ϵ_{cap}. (c-f) Calculation results analogous to panels a-c, but for a t_2 = 15 nm-thin PMMA subsurface layer. (a-f) The horizontal dashed line indicates ϵ_{PS} which is used in the manuscript. Gray areas indicate peak heights $\varphi_4^{\max} < 5°$, which we consider to be below the detection threshold. The figure shows that: (*1*) for each capping layer permittivity ϵ_{cap} and PMMA layer thickness t_2 the nano-FTIR peak positions shift to lower frequencies (red-shift) when the depth d_2 of a subsurface layer increases. (*2*) The red-shift is stronger when capping layer permittivity increases. (*3*) With increasing permittivity of the capping layer the peak heights reduce, which in turn reduces the depths d_2 at which a nano-FTIR peak can be practically detected. (*4*) Most interesting and important, the peak height ratios $C = \varphi_4^{\max}/\varphi_3^{\max}$ observed for all capping layer permittivities and both thicknesses t_2 decreases as the depth d_2 increases, corroborating the robustness as criterium for distinguishing surface and subsurface layers.

Supplementary References

1. Ocelic, N. Quantitative near-field phonon-polariton spectroscopy. (Technische Universität München, 2007).

2. Cvitkovic, A., Ocelic, N. & Hillenbrand, R. Analytical model for quantitative prediction of material contrasts inscattering-type near-field optical microscopy. *Opt. Express* **15**, 8550–8565 (2007) https://doi.org/10.1364/OE.15.008550.

3. Jackson, J. D. *Classical Electrodynamics*. (John Wiley & Sons, 2007).

4. Novotny, L. & Hecht, B. *Principles of Nano-Optics*. (Cambridge, 2006).

5. Nolting. *Grundkurs theoretische Physik. Bd.3: Elektrodynamik*. vol. 3.

6. Aizpurua, J., Taubner, T., Javier Garcia de Abajo, F., Brehm, M. & Hillenbrand, R. Substrate-enhanced infrared near-field spectroscopy. *Optics Express* (2008) https://doi.org/10.1364/OE.16.001529.

7. Fei, Z. *et al.* Infrared Nanoscopy of Dirac Plasmons at the Graphene–SiO2 Interface. *Nano Lett.* **11**, 4701–4705 (2011) https://doi.org/10.1021/nl202362d.

8. Hauer, B., Engelhardt, A. P. & Taubner, T. Quasi-analytical model for scattering infrared near-field microscopy on layered systems. *Optics Express* **20**, 13173 (2012) https://doi.org/10.1364/OE.20.013173.

9. McLeod, A. S. *et al.* Model for quantitative tip-enhanced spectroscopy and the extraction of nanoscale-resolved optical constants. *Phys. Rev. B* **90**, 085136 (2014) https://doi.org/10.1103/PhysRevB.90.085136.

10. Jiang, B.-Y., Zhang, L. M., Castro Neto, A. H., Basov, D. N. & Fogler, M. M. Generalized spectral method for near-field optical microscopy. *Journal of Applied Physics* **119**, 054305 (2016) https://doi.org/10.1063/1.4941343.
